# Dehydration-enhanced ion-pore interactions dominate anion transport and selectivity in nanochannels

**DOI:** 10.1126/sciadv.adf8412

**Published:** 2023-07-07

**Authors:** Chenghai Lu, Chengzhi Hu, Zhibin Chen, Peiyao Wang, Fan Feng, Guangzhi He, Fuyi Wang, Yanyan Zhang, Jefferson Zhe Liu, Xiwang Zhang, Jiuhui Qu

**Affiliations:** ^1^ State Key Laboratory of Environmental Aquatic Chemistry, Research Center for Eco-Environmental Sciences, Chinese Academy of Sciences, Beijing 100085, China.; ^2^ University of Chinese Academy of Sciences, Beijing 100049, China.; ^3^ Department of Mechanical Engineering, The University of Melbourne, Parkville, VIC 3010, Australia.; ^4^ Beijing National Laboratory for Molecular Sciences, CAS Key Laboratory of Analytical Chemistry for Living Biosystems, Institute of Chemistry, Chinese Academy of Sciences, Beijing 100190, China.; ^5^ UQ Dow Centre for Sustainable Engineering Innovation, School of Chemical Engineering, The University of Queensland, St. Lucia, QLD 4072, Australia.

## Abstract

State-of-the-art ion-selective membranes with ultrahigh precision are of significance for water desalination and energy conservation, but their development is limited by the lack of understanding of the mechanisms of ion transport at the subnanometer scale. Herein, we investigate transport of three typical anions (F^−^, Cl^−^, and Br^−^) under confinement using in situ liquid time-of-flight secondary ion mass spectrometry in combination with transition-state theory. The operando analysis reveals that dehydration and related ion-pore interactions govern anion-selective transport. For strongly hydrated ions [(H_2_O)_n_F^−^ and (H_2_O)_n_Cl^−^], dehydration enhances ion effective charge and thus the electrostatic interactions with membrane, observed as an increase in decomposed energy from electrostatics, leading to more hindered transport. Contrarily, weakly hydrated ions [(H_2_O)_n_Br^−^] have greater permeability as they allow an intact hydration structure during transport due to their smaller size and the most right-skewed hydration distribution. Our work demonstrates that precisely regulating ion dehydration to maximize the difference in ion-pore interactions could enable the development of ideal ion-selective membranes.

## Teaser

The important role of dehydration in anion confined transport was revealed using in situ liquid ToF-SIMS.

## INTRODUCTION

High-precision ion-selective membranes with subnanometer pores have attracted increasing interest due to their widespread applications in water purification, energy conservation, and resource recovery (*1*, *2*). Understanding the mechanisms for ion transport in confined nanochannels, typically involving dehydration (*3*–*5*) and ion-pore interactions (*5*–*7*), is crucial to the development of high-performance ion-selective membranes (*2*, *8*). Despite extensive efforts on experimental investigation of dehydration and the related interplays between ions and functional groups, ion transport behavior under confinement has not been fully understood because of the lack of operando characterization techniques (*4*, *9*, *10*).

Differing ion dehydration behavior at the water-membrane interface dictates ion transport or, more particularly, the ion-ion selectivity (*3*). According to traditional ion dehydration theory, the dehydration of an ion is influenced by various bulk solvation properties, e.g., hydration energy (*11*, *12*), hydration size (*12*), and effective charge (*13*, *14*). However, these parameters fail to explain the ion transport behavior because of the unpredictable hydration conformation and distribution resulting from anisotropic solvation (*10*, *11*). Previous studies have predominantly focused on observational reasoning between parameters involved in transport and hydration properties, for instance, permeability and hydration radii (*15*), which are unfortunately insufficient to elucidate the thermodynamics/kinetics of molecular interactions during ion transport. Transition-state theory (TST) for transmembrane permeation, which links certain selectivity trends to the apparent transmembrane energy barriers, can partly evaluate the role of dehydration in transport (*9*, *13*, *15*–*17*). Nevertheless, this theory so far has failed to quantify the energy requirements of dehydration and other related interactions at different stages of transport because of the inability to provide dynamic information regarding the hydration structure of ion during transport in nanochannels (*4*). In addition, for this reason, whether the partitioning into the pore entrance, which involves dehydration, or intrapore diffusion dominates the ion transport remains a subject of debate (*16*, *18*).

Another open question is how the ion-pore interactions affect ion transport and selectivity, in particular, after dehydration (*8*, *16*, *19*). Typical ion-pore interactions include electrostatic repulsion (e.g., the Donnan effect) (*2*), viscous effect (e.g., chemical affinity) (*8*), and frictional interactions (e.g., physical collisions) (*8*). Given that such interactions are inextricably linked to the size and effective charge of an ion (*6*, *13*, *20*, *21*), dehydration will influence ion-pore interactions, consequently affecting ion selectivity. For example, biological ion channels can achieve ultrahigh ion selectivity through rational combination of dehydration and ion-channel interactions (*7*, *21*, *22*). The influence of an ion after dehydration on ion-pore interactions, however, remains elusive, because of the inability to capture changes in hydration structure (*4*) and predict the magnified ion-pore interactions under confinement (*23*).

Collectively, the dehydration mechanism of ion transport and its effect on ion selectivity are still poorly understood. Hence, it is necessary to elucidate the correlation between ionic properties and dehydration by dynamic hydration structure characterization (*4*). In this work, for this purpose, three typical anions (F^−^, Cl^−^, and Br^−^) with identical charge and minor chemical difference, which are ubiquitous in biological systems and water purification, were selected as model ions. The dehydration behavior of the anions when passing through confined nanochannels in polyamide nanofiltration membranes was investigated using in situ liquid time-of-flight secondary ion mass spectrometry (ToF-SIMS), which enables operando discrimination of hydration states. Coupling operando characterization, TST calculation, and computational simulations, we found that the charge density dictates the ion hydration distribution and hence the confinement-induced dehydration. The quantification of the energy barriers contributed from steric size and electrostatics highlights how dehydration-enhanced ion-pore interactions can appreciably increase anion selectivity. Specifically, the partial dehydration of strongly hydrated anions, i.e., (H_2_O)_n_F^−^ and (H_2_O)_n_Cl^−^, was found to contribute higher energy barriers from dehydration-enhanced electrostatic interactions, which leads to more hindered transport, while (H_2_O)_n_Br^−^ with a softer hydration shell and the most right-skewed hydration distribution exhibits lower energy barriers by maintaining an intact hydrated structure through the polymeric nanochannels. Our findings shed light on a dehydration-driven mechanism dominating ion selectivity in confined transport, which reveals that the precise matching between size effect (i.e., dehydration) and functional groups (i.e., ion-pore interactions) is an important guideline for the future development of ion-selective membranes.

## RESULTS

### In situ characterization of dehydration during ion transmembrane transport

**Fig. 1.**
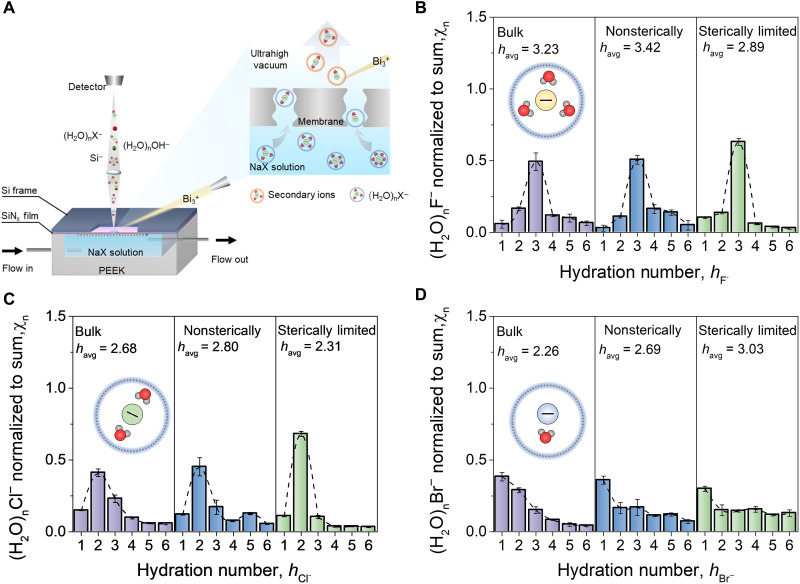
In situ characterization of *h*_X^−^_ distribution during transmembrane ion transport. (**A**) Schematic diagram showing in situ liquid ToF-SIMS analysis combined with the microfluidic nanofiltration platform. The detailed procedure of this method is given in Materials and Methods. (**B** to **D**) Variation of *h*_X^−^_ distributions before and after filtration of 10 mM NaF, NaCl, and NaBr, respectively. The left panel of each figure represents the *h*_X^−^_ distributions in the bulk solution (i.e., before filtration), the middle panel represents the *h*_X^−^_ distributions after filtration by nonsterically limited membrane (NF 800), and the right panel represents the *h*_X^−^_ distributions after filtration by sterically limited membrane (NF 200). *h*_avg_ denotes the weighted average strongly bound water number of the corresponding *h*_X^−^_ distributions only for mathematical comparison. The dotted lines in all panels are provided to guide the eye. Error bars represent SDs.

The operando characterization of the dehydration of the three monovalent anions (F^−^, Cl^−^, and Br^−^) during transmembrane transport was conducted on an in situ liquid ToF-SIMS microfluidic filtration platform with two polyamide nanofiltration membranes, NF 800 and NF 200 (Fig. 1A). The hydration distributions (*h*_X^−^_) of the anions were further calculated by normalizing the ToF-SIMS peak intensities of individual hydrates to the total hydrated ion mass spectrum (note S2, details in the Supplementary Materials). In bulk solutions (before filtration), the *h*_X^−^_ shows a chi square–like distribution, and the distribution gradually turns to right-skewed as the ionic radii increase. The dominant hydrates of the three anions (representing the hydrated species with highest proportion in *h*_X^−^_) are (H_2_O)_3_F^−^, (H_2_O)_2_Cl^−^, and (H_2_O) Br^−^, respectively. The observed dominant hydrates were lower than those measured by molecular dynamics (MD) simulation but were in good agreement with the hydration number obtained from theoretical extractions based on bulk properties. This indicates that *h*_X^−^_ determined by ToF-SIMS refers primarily to water molecules that are strongly bound to the ion by its Coulombic field and moved along with the ion in the solution (detailed discussion in note S2). One can see that the charge density dictates an ion’s hydration size as the observed average strongly bound water number (*h*_avg_) shows the order (H_2_O)_n_F^−^ > (H_2_O)_n_Cl^−^ > (H_2_O)_n_Br^−^ (Fig. 1, B to D). Given that the hydrated ions all have the same charge of −1, the smaller the ionic radii, the less hydrogen-bond networks need to be broken to hydrate ions, which is energetically favorable for the capture of more bound waters (*24*–*26*).

During the filtration of the ion solutions by NF 800, a loose nanofiltration membrane (denoted as nonsterically limited membrane), no apparent dehydration was observed, as the proportion (χ_n_) of the larger hydrates and *h*_avg_ even slightly increased (Fig. 1, B to D, middle). Conversely, dehydration occurred in NF 200, a tighter nanofiltration membrane (denoted as sterically limited membrane), for the larger (H_2_O)_n_F^−^ and (H_2_O)_n_Cl^−^, as evidenced by the decrease in their *h*_avg_ (Fig. 1, B and C, right). Coinciding with the observed decrease in *h*_avg_, the proportion of hydrates larger than the dominant form (i.e., χ_F^−^,*n*>3_ and χ_Cl^−^,*n*>2_) notably diminished after the filtration by NF 200 (Fig. 1, B and C, right). This hydration change signifies that the (H_2_O)_n_F^−^ and (H_2_O)_n_Cl^−^ must partially lose their solvation shell to reduce their effective size to pass through the tighter membrane. In contrast, (H_2_O)_n_Br^−^ has a similar *h*_Br^−^_ distribution after being filtered by NF 200 and NF 800 (Fig. 1D, middle and right). That is, no detectable dehydration occurred for (H_2_O)_n_Br^−^ even under confinement, which might be attributed to *h*_Br^−^_ having the most right-skewed distribution in the bulk (Fig. 1D, left) and its “softer” hydration shell. Compared with the (H_2_O)_n_F^−^ and (H_2_O)_n_Cl^−^, most (H_2_O)_n_Br^−^ species have a smaller size in the bulk. The smaller size of these hydrated species consequently allows (H_2_O)_n_Br^−^ to permeate through membranes more easily. Besides, the lower hydration enthalpy of (H_2_O)_n_Br^−^ (table S1) contributes to a smaller and softer hydration shell, which may make the rearrangement of (H_2_O)_n_Br^−^ hydration shell more energetically favorable compared to the dehydration for the traverse across nanochannels (*12*, *27*).

The pore sizes of the two nanofiltration membranes and the estimated hydrodynamic radii of hydrates (*r*_H−X^−^_) (*28*) follow the order: μ_p,NF800_ (0.48 nm) > *r*_H−F^−^_ (0.352 nm) > *r*_H−Cl^−^_ (0.332 nm) > *r*_H−Br^−^_ (0.330 nm) > μ_p,NF200_ (0.30 nm) (fig. S1 and table S1), which confirms that dehydration is attributed to the size compatibility between the hydrates and nanochannels. MD simulation was conducted to verify the change in hydration during (H_2_O)_n_X^−^ transport through polyamide nanochannels (fig. S2, details in the Supplementary Materials). As shown in fig. S3, the values of *h*_F^−^_ and *h*_Cl^−^_ markedly decrease after the hydrates enter into the smaller nanochannel with *r* ≈ 0.3 nm (comparable with μ_p_ of NF 200), confirming the confinement-induced partial dehydration of (H_2_O)_n_F^−^ and (H_2_O)_n_Cl^−^. Contrastingly, no obvious dehydration was observed for any of the anions in the larger nanochannel (*r* ≈ 0.5 nm, comparable with μ_p_ of NF 800), evidenced by that the *h*_X^−^_ of these ions in the bulk and the 0.5-nm channel being very close (fig. S3). (H_2_O)_n_Br^−^ maintains its hydration number even in the 0.3-nm channel (fig. S3, C and F), in good agreement with the operando characterization—although the *r*_H−Br^−^_ is slightly larger than the pore size. We speculate that the (H_2_O)_n_Br^−^ might enter the sterically limited nanochannel by rearrangement of hydration shell rather than dehydration because their hydration layer is smaller and softer than (H_2_O)_n_F^−^ and (H_2_O)_n_Cl^−^ (more detailed discussion is shown in note S3).

The structures of various (H_2_O)_n_X^−^ were further obtained by ab initio density functional theory (DFT) (fig. S4). For (H_2_O)_n_Cl^−^ and (H_2_O)_n_Br^−^, Cl^−^ and Br^−^ are located on the surface of the bound water clusters, thus favoring a surface structure (*29*, *30*), while (H_2_O)_n_F^−^ favors a semisurface structure due to the stronger F^−^-water interaction (*31*, *32*). Since the water molecules in the semisurface structure are more evenly distributed around the anion, the (H_2_O)_n_F^−^ are relatively more sensitive to confinement in comparison with the lopsided surface structures of (H_2_O)_n_Cl^−^ and (H_2_O)_n_Br^−^ where the water molecules are concentrated on one side of the anion (fig. S4, more detailed discussion in note S4). Despite the similar hydrodynamic radii and hydration shapes of (H_2_O)_n_Cl^−^ and (H_2_O)_n_Br^−^, their dehydration behavior is quite different. Because of the more right-skewed *h*_Br^−^_ distribution in the bulk and the softer hydration shell, (H_2_O)_n_Br^−^ is thus more likely to maintain an integrated hydration shell. These simulation results coincide well with the *h*_X^−^_ distribution obtained by in situ liquid ToF-SIMS. Overall, the results indicate that (i) (H_2_O)_n_F^−^ and (H_2_O)_n_Cl^−^ are partially dehydrated during transport through the sterically limited NF 200 and (ii) no dehydration occurs in the nonconfined NF 800 membrane for the three hydrates.

### The critical role of dehydration during ion partitioning into nanochannels

**Fig. 2.**
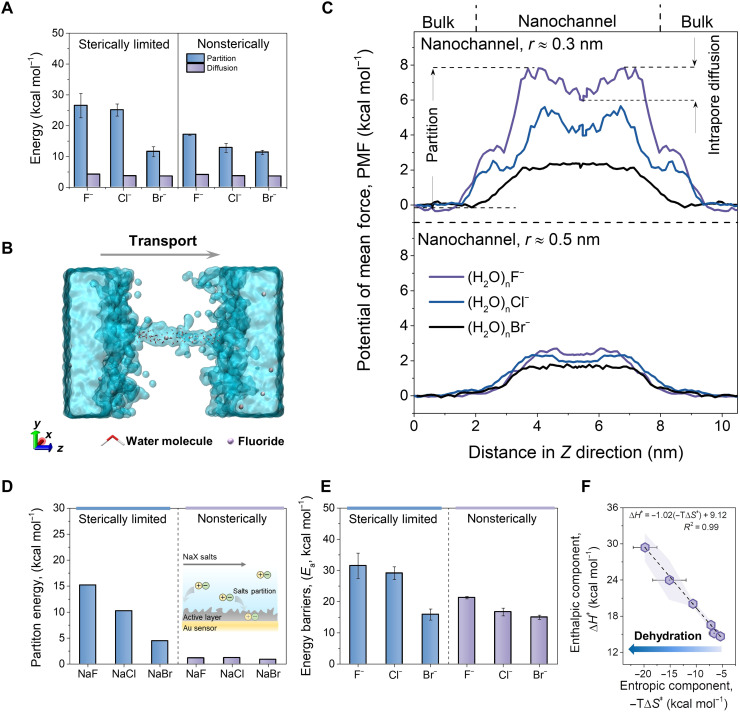
Elucidation of the critical role of dehydration during ion partition using TST. (**A**) The apparent energy for hydrated anions entering the polyamide membranes (i.e., partitioning) and diffusing inside the polyamide membranes (i.e., intrapore diffusion) obtained by the hindered transport theory in combination with TST. (**B**) Cross-sectional illustration of the simulation platform. The cyan part represents the water-filled reservoirs with NaX solution (X = F, Cl, and Br) connected by a polyamide nanochannel. The simulated bulk polyamide was omitted for improved clarity (more details in the Supplementary Materials, fig. S2). (**C**) MD simulation of the change in the PMF during ion transport across polyamide nanochannels with different size. (**D**) Apparent energy for NaX salts partitioning into the active layer of membranes quantified by QCM. (**E**) Measured overall apparent transmembrane energy barriers (*E*_a_) for anion transport through polyamide membranes. (**F**) Correlation between measured energy of enthalpic component (Δ*H*^‡^) and entropic component (−*T*Δ*S*^‡^) for anions at 298.15 K, showing an EEC in anion transport. The data were collected from the Δ*H*^‡^ and −*T*Δ*S*^‡^ of the three anions (i.e., F^−^, Cl^−^, and Br^−^) during transport in NF 200 and NF 800. The shaded areas represent the SDs of the enthalpic component (Δ*H*^‡^). The blue arrow indicates that stronger dehydration is accompanied by higher entropic compensation.

Ion transport through nanochannels occurs by two sequential steps: partitioning into the nanochannels and intrapore diffusion. To examine the rate-limiting transport step, we determined the apparent energies for the three anions to partition (i.e., *E*_partition_) and diffuse (i.e., *E*_diffusion_) by using the hindered transport theory in combination with TST. As is shown in Fig. 2A, *E*_partition_ is much higher than the *E*_diffusion_, showing that partitioning into the nanochannels contributes higher resistance for the permeation of solvated anions through polyamide nanochannels. We then calculated the potential of mean force (PMF) profile of monovalent anion transport across polymeric nanochannels using an MD simulation platform (Fig. 2B) to further understand the transport process of the anions through polyamide nanochannels. The energy for ion transport is reflected by the increase in the PMF when solvated ions partition into the pore entrance (*Z* ≈ 2 nm) (Fig. 2C). A saddle point following partition indicates that ions first undergo energetically favorable transport after entering the nanochannels, which is followed by the PMF of all ions increasing again because of the energy requirement of ion-pore interactions in intrapore diffusion. The simulated *E*_partition_ is also higher than *E*_diffusion_ (fig. S5), which is in good agreement with the observation by TST. A quartz crystal microbalance (QCM) was then used to quantify the apparent partition energy of sodium-based salts (that is, NaF, NaCl, and NaBr) (Fig. 2D, details in the Supplementary Materials). The measured apparent partition energy trend mirrors *E*_partition_, which experimentally evidences the partitioning behavior of the three anions observed by hindered transport theory and MD simulations. The partition-dominated transport of the three anions in polyamide membrane could be due to the negatively charged characteristics of polyamide membranes under our experiment conditions. The electrostatic repulsion between the anions and the negatively charged pore wall with ionized carboxyl groups leads anions to move away from the pore wall, which may reduce the influence of ion-pore interactions on intrapore diffusion. The negative nanochannels are thus conducive to intrapore diffusion of the three anions (fig. S6, details in the Supplementary Materials). The dehydration and electrostatic repulsion at the solution-membrane interface further result in relatively higher *E*_partition_ of the anions.

The energy contribution during ion transport was further studied using TST. The strong correlation between the overall apparent transmembrane energy barriers (*E*_a_) and *h*_X^−^_ solvated size, i.e., *E*_a_(F^−^) > *E*_a_(Cl^−^) > *E*_a_(Br^−^) (Fig. 2E), coincides well with the PMF trend in MD simulation. The more substantial increase of *E*_a_ for (H_2_O)_n_F^−^ and (H_2_O)_n_Cl^−^ in NF 200 (Fig. 2E) indicates a more intense size effect. On the basis of the TST for transmembrane permeation, the energy of ion permeation can be broken down into enthalpic (∆*H^‡^*) and entropic (−*T*∆*S${}^{‡}$*) components (note S9, details in the Supplementary Materials) to highlight the molecular features of ion transport. Mechanisms in membrane permeation related to the enthalpic effect, such as dehydration and electrostatic repulsion, can contribute to ∆*H${}^{‡}$*, while −*T*∆*S${}^{‡}$* is associated with steric effects (*9*). As shown in fig. S7, the value of ∆*H${}^{‡}$* is similar to *E*_a_, implying that the hindrance of anion partitioning into nanochannels is mainly from enthalpy-related dehydration and charge repulsion between anions and negative pore entrance of polyamide membrane. Moreover, anions with higher hydration enthalpy experienced stronger ∆*H${}^{‡}$*, i.e., ∆*H${}^{‡}$*(F^−^) > ∆*H${}^{‡}$*(Cl^−^) > ∆*H${}^{‡}$*(Br^−^), providing further support for the supposition that dehydration plays an important role in the transmembrane hinderance. Note that the ∆*H${}^{‡}$* for (H_2_O)_n_Br^−^ appears to be independent of the pore size (fig. S7), meaning that soft hydrates could maintain their hydrated structure during transport.

Another important evidence of the critical role of dehydration during ion partitioning into nanochannels is the entropic compensation mechanism. The three anions showed negative entropic components (−*T*∆*S${}^{‡}$*) with the order of −*T*∆*S${}^{‡}$*(F^−^) < −*T*∆*S${}^{‡}$*(Cl^−^) < −*T*∆*S${}^{‡}$*(Br^−^) (fig. S8), which mirrors well with the trend of the energy decline observed in the simulated PMF after entering nanochannels (Fig. 2C). This indicates that the anions are compensated by entropy after partitioning into the nanochannels. Furthermore, the −*T*∆*S${}^{‡}$* term is inversely proportional to ∆*H${}^{‡}$*, where higher −*T*∆*S${}^{‡}$* is accompanied by lower ∆*H${}^{‡}$* and vice versa (Fig. 2F), namely, entropy-enthalpy compensation (EEC). Ion solvation in the bulk is entropically unfavorable (that is, ∆*S${}^{‡}$* < 0; table S1), while the dehydration thus takes the reverse (*10*). Dehydration would release the restricted hydrated structure to favorably transport anions, which subsequently produces entropy (∆*S${}^{‡}$* > 0). Anions with more negative −*T*∆*S${}^{‡}$* (i.e., stronger dehydration) experience relatively higher enthalpic resistance from dehydration. Overall, the results highlight the dominant role of partitioning in anion transport across polymeric nanochannels, which contributes the energy barriers for hydrated anions from enthalpy-related dehydration and charge repulsion to govern ion transport.

### Dehydration enhances ion-pore interactions

**Fig. 3.**
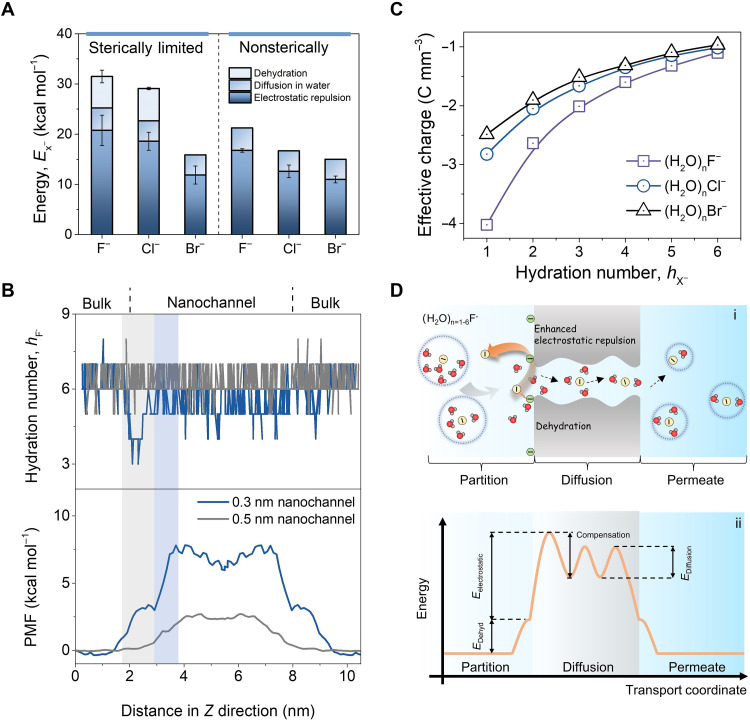
Illustration of dehydration-enhanced electrostatic interactions on ions during transmembrane transport. (**A**) Energy contributed to apparent energy barriers by various steps throughout the transmembrane transport. (**B**) Variation of hydration number and PMF of (H_2_O)_n_F^−^ in polyamide nanochannels with different sizes. The gray shading indicates that the change in hydration number at the pore entrance is accompanied by an increase in PMF. The blue shading indicates the change in energy following dehydration. (**C**) DFT calculation of effective charge for hydrated anions with various hydration numbers. (**D**) Schematic diagram showing (i) ion transport and (ii) energy profile of strongly hydrated anions for elucidating the mechanisms of dehydration-enhanced electrostatic interactions. Hydrates permeate across the membrane as a result of partitioning into the active layer and diffusion through the active layer. For strongly hydrated ions [e.g., (H_2_O)_n_F^−^] under confinement, these larger hydrates would partially dehydrate to reduce their effective size for permeation, but this simultaneously enhances the electrostatic repulsion between negative carboxyl groups and dehydrated anions, contributing higher energy barriers. Yellow and green balls represent the fluoride (F^−^) and negative carboxyl, respectively.

The main hindrance of anions during transport in polyamide nanofiltration membrane is from size and charge effect according to the well-accepted anion exclusion mechanisms (*13*). The energy barriers of anion transport can thus be divided into three aspects: (i) dehydration ($\text{Δ}H_{\mathrm{dehyd}}^{‡}$), (ii) ion diffusion in water (*E*_w_), and (iii) anion-functional group electrostatic repulsion (*E*_ele_). On the basis of the data from in situ liquid ToF-SIMS and TST, the contribution of three aspects was then quantified. Unexpectedly, *E*_ele_ is the dominant contributor rather than dehydration (Fig. 3A). Although $\text{Δ}H_{\mathrm{dehyd}}^{‡}$ is not as predominant as expected, partial dehydration leads to stronger electrostatic repulsion for (H_2_O)_n_F^−^ and (H_2_O)_n_Cl^−^ (i.e., higher *E*_ele_ in NF 200; Fig. 3A). Accordingly, (H_2_O)_n_Br^−^ shows similar *E*_ele_ in both the membranes due to the intact hydrated structure during transport. The lower $\text{Δ}H_{\mathrm{dehyd}}^{‡}$ that we observed could be due to the flexible nature of polyamide membrane (*10*) and compensation for dehydration barriers by ion-dipole interactions between anion hydrates and polar groups of polyamide membrane (*20*, *27*). The relative influence between dehydration and ion-pore interactions is dependent on the specific membrane structures. The dominant role of *E*_ele_ in our study could be attributed to the flexible structures with negative carboxyl of polyamide membrane (*10*). By comparing the variation trend between *h*_X^−^_ and PMF of the (H_2_O)_n_F^−^ (summarized in the Fig. 3B), we can conclude that for (H_2_O)_n_F^−^, ion dehydration dominates from 2 to 3 nm in the polyamide model, whereas the ion-pore interactions dominate from 3 to 4 nm. The corresponding contribution is about 3 and 5 kcal mol^−1^. This is consistent with our experimental results, i.e., ion-wall interaction plays a more substantial contribution in the studied polyamide membranes. The simulated energy contribution from ion-pore interactions of (H_2_O)_n_F^−^ and (H_2_O)_n_Cl^−^ in the 0.3-nm nanochannel was higher than that in the 0.5-nm nanochannel, which is consistent with our experiment results from in situ liquid ToF-SIMS and TST.

We further analyzed the effective charges of (H_2_O)_n_X^−^ with DFT calculations to investigate the mechanisms of the dehydration-enhanced ion-pore interactions (note S10, details in the Supplementary Materials). The effective charges of the anions increase notably with the decrease in the number of bound water molecules (Fig. 3C). This is because dehydration can reduce the charge shielding effect of solvated waters around the central ion (*13*, *33*, *34*). Fluoride, with the smallest ionic radius, has remarkably higher effective charge than chloride and bromide. Besides, dehydration is favorable for shortening the distance (*r*_0_) between hydrates and the negative carboxyl functional groups on the surface of polyamide nanochannels (fig. S9). Since electrostatic repulsion is correlated to effective charges and $\frac{1}{r_{0}^{2}}$ (*35*), shedding solvated water would therefore enhance the charge repulsion by negative carboxyl groups. The partially dehydrated (H_2_O)_n_F^−^ thus are more strongly repulsed, resulting in higher energy barriers. The traverse speed of the three anions was simulated, which follows the sequence (H_2_O)_n_F^−^ < (H_2_O)_n_Cl^−^ < (H_2_O)_n_Br^−^ (fig. S10), supporting the proposed mechanisms.

The results suggest that anion transport in polyamide membranes is dominated by ∆*H${}^{‡}$* during ion partitioning at the pore entrance. Dehydration would reinforce the electrostatic repulsion between partially dehydrated hydrates and membranes. Figure 3D and fig. S11 illustrate the mechanisms and corresponding energy barrier profiles of the enhanced charge repulsion driven by dehydration in polymeric nanochannels, highlighting the role of the nanochannel size and electrostatics. Specifically, strongly hydrated anions larger than the membrane pores (i.e., sterically limited) tend to sterically dehydrate, leading to enhanced electrostatic repulsion and higher energy barriers [e.g., (H_2_O)_n_F^−^; Fig. 3D]. Conversely, softer solvated anions maintain an intact hydration layer by shell rearrangement, resulting in less hindered transport and lower energy barriers [e.g., (H_2_O)_n_Br^−^; fig. S11].

### Dehydration-enhanced ion-pore interactions dominate ion selectivity in membrane separation

**Fig. 4.**
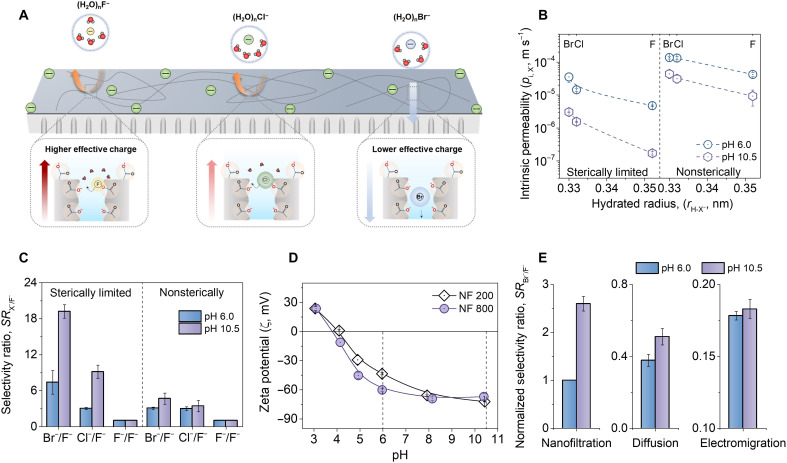
Ion transport and selectivity of nanoporous polymeric nanochannels. (**A**) Schematic diagram showing dehydration-enhanced electrostatic interactions on hydrates. Partially dehydrated anions with higher effective charge [e.g., (H_2_O)_n_F^−^] could interact more closely with the ionized carboxyl groups and are thus repulsed intensely by the membrane. Red arrows represent the intensity of electrostatic repulsion and dominant transport direction of hydrated anions, where a lighter color represents stronger repulsion. (H_2_O)_n_Br^−^, which maintains an intact hydrated shell during permeation and interacts weakly with the ionized carboxyl groups thus has greater permeability across the membrane (light blue arrow). (**B**) Intrinsic permeability of monovalent anions in nanofiltration under different pH. (**C**) Anion selectivity ratio (*SR*_X^−^/F^−^_) of the two polyamide nanofiltration membranes (i.e., sterically limited NF 200 and nonsterically limited NF 800) under different pH. (**D**) Zeta potential change with respect to pH for NF 200 and NF 800 membranes. (**E**) Normalized selectivity ratio between Br^−^ and F^−^, i.e., *SR*_Br^−^/F^−^_, under different driving forces in NF 200. All selectivity ratios were normalized on the basis of the *SR*_Br^−^/F^−^_ of NF 200 in nanofiltration at pH 6.0.

Our findings highlight the important role of dehydration and related ion-pore interactions in ion transport (Fig. 4A). We further investigated how this mechanism governs ion transport and selectivity in membrane separation by conducting pressure-driven nanofiltration experiment (fig. S12A). The experimental results show that the anion permeability and selectivity show a strong dependence on the hydrated size or apparent energy barriers (Fig. 4, B and C). Softer hydrates have higher ion permeability (*p*_i,X^−^_), i.e., *p*_i,F^−^_ < *p*_i,Cl^−^_< *p*_i,Br^−^_ as expected. The size effects play an important role in the selectivity as a higher selectivity ratio (*SR*_X^−^/F^−^_) was found in the sterically limited NF 200 (Fig. 4C), where *SR*_Br^−^/F^−^_ reached 7.39. Anion permeability showed less difference in nonsterically limited NF 800 membranes, with *SR*_Br^−^/F^−^_ and *SR*_Cl^−^/F^−^_ near 3.00 (Fig. 4, B and C, right).

The sensitivity to a subnanometer environment is different for the three anions, which is determined by their hydration characteristics (Fig. 1). The stronger electrostatic interactions of the dehydrated (H_2_O)_n_F^−^ make it more hindered compared to (H_2_O)_n_Cl^−^ and (H_2_O)_n_Br^−^ (Fig. 4A). The higher value of *SR*_Br^−^/F^−^_ is accordingly justified. On the basis of the proposed mechanisms of the dehydration-enhanced ion-pore interactions, the electrostatic repulsion of (H_2_O)_n_F^−^ would be more pronounced with the enhancement of the membrane charge due to its stronger dehydration. When pH was then increased to 10.5, the fully ionized carboxyl groups gave both membranes more negatively charged surface (Fig. 4D and fig. S13). A notable decrease in *p*_i,F^−^_ in NF 200 (Fig. 4B, left) subsequently raised the *SR*_Br^−^/F^−^_ from 7.39 to 19.22 (Fig. 4C, left). For the nonsterically limited NF 800, however, inconspicuous improvement of *SR*_X^−^/F^−^_ was observed. This stems from the almost identical decrease in permeability for all anions in NF 800 (Fig. 4B, right). These trends of anion intrinsic permeability and *SR*_X^−^/F^−^_ remain the same after accounting for concentration polarization (fig. S14). In other words, electrostatic interactions can substantially improve the ion selectivity when driven by dehydration.

To eliminate the deviation from the accurate transport behavior of hydrates induced by convective flow and concentration polarization in pressure-driven nanofiltration, we therefore investigated the transport of the three anion hydrates driven by concentration gradient (diffusion; fig. S12B) and electric field (electromigration; fig. S12C). A consistent apparent energy barrier dependence of ion selectivity was observed after excluding the influence of convective flow and concentration polarization (*SR*_Br^−^/F^−^_ > *SR*_Cl^−^/F^−^_ > *SR*_F^−^/F^−^_; fig. S15). The improvement of ion selectivity by dehydration-enhanced electrostatic repulsion still exists in diffusion and electromigration, as *SR*_X^−^/F^−^_ was improved in NF 200 at higher pH (Fig. 4E and fig. S15, B and D). Notably, this enhancement in diffusion and electromigration was far less effective than that in the nanofiltration (Fig. 4E). This could be attributed to the higher extent of dehydration in nanofiltration, which subsequently led to the substantial influences of charge repulsion on transmembrane hydrates (*14*). Our results demonstrate that anion selectivity in polyamide membrane is primarily governed by the ion-pore interactions after dehydration. A well-tailored pore structure to precisely regulate the hydrated size by dehydration to maximize the difference in ion-pore interactions can be an effective design guideline for the development of ideal ion-selective membranes.

## DISCUSSION

Despite intense efforts to resolve the selectivity mechanisms governing ion transport (*9*, *10*, *20*, *21*), the impacts of dehydration and related ion-pore interactions on the ion transport behavior at the subnanometer scale are not yet fully understood. We herein elucidate how dehydration governs ion selectivity by leveraging in situ liquid ToF-SIMS and TST. Our study highlights the influence of enhanced electrostatic interactions due to dynamic change in the hydration structure brought about by dehydration on ion transport and selectivity. Notably, the proposed mechanism can be extended to other ion-pore interactions, e.g., chemical affinity and coordination interactions, because they are closely related to the hydrated ion size. This would be an important step forward in understanding uncommon transport phenomena under confinement in many industrial and biomedical applications (e.g., ionic pumps, desalination, and biosensing). Establishing this methodology can provide direct and deeper insights into hydrated structure transformations during transmembrane transport, which would open up a promising avenue for nanofluidics investigatory.

According to analysis of the dynamic changes in the hydration states and apparent energy barriers in our study, to selectively recover specific ions, one should first determine the rate-limiting step for the transmembrane transport. For instance, the apparent energy barriers for the permeation of anions through negative polymeric nanochannels are governed by partitioning at the pore entrance. In this case, the pore size and functional groups at the pore entrance should be carefully designed to maximize ion-pore interactions by dehydration of the nontarget ions, hence increasing the apparent partitioning energy barriers. In addition, the selection of appropriate intrapore functional groups might facilitate the permeation of target ions through a compensatory mechanism. This design framework echoes the biological ion channel, which regulates the optimal size of the traversing hydrates by dehydration to favorably interact with the binding sites to achieve ultrafast transport (fig. S16) (*36*). The KcsA channels, for instance, let 10^4^ K^+^ ions pass for every Na^+^ ion (*5*, *36*), yet the selectivity factor of the traditional thin film composite is no more than 20 (*13*, *15*, *37*). This could be attributed to the less sophisticated structure of conventional membrane materials—polyamide-based membranes have a broad pore size distribution and nonspecific carboxyl or amino groups, which limit the ability to increase the energy barriers difference between ions. Therefore, we believe that the studied dehydration- and ion-pore interaction–based transport mechanisms have great potential for the design of selective membrane materials. Future investigations should further focus on the matching between uniform pore structures and specific functionality in membrane materials. Overall, our study provides two potential guidelines for tailoring ion-selective membranes: (i) carefully selected functional groups on the surface or in the interior (depending on the dominant steps of energy barriers) based on intrinsic differences in the nature of the solutes and (ii) further tailoring the pore structure to optimally match specific interactions between ions and functional groups by dehydration.

## MATERIALS AND METHODS

### Chemicals and materials

Deionized (DI) water (>18.2 megaohms·cm) produced from a Milli-Q ultrapure water purification system (Millipore Milli-Q) was used for preparing electrolyte solutions. Sodium fluoride (NaF, ≥99.9%), sodium chloride (NaCl, ≥99.9%), sodium bromide (NaBr, ≥99.9%), sodium hydroxide (NaOH, ≥99.9%), *N,N*-dimethylformamide (≥99.5%), ethylene glycol (C_2_H_6_O_2_, ≥99%), diethylene glycol (C_4_H_10_O_3_, ≥99%), polyethylene glycol (≥99%, MWs of 200, 400, 600, and 800 Da), nitric acid (HNO_3_, 65 to 68%), hydrochloric acid (HCl, 36 to 38%), and silver nitrate (AgNO_3_, ≥99%) were purchased from Sinopharm Chemical Regent Co. Ltd., China; 0.1 M NaOH and HCl was used to adjust the solution pH in experiments. The silicon nitride (SiN_x_) film window was obtained from Norcada Inc. (Edmonton, Canada). The QCM Au sensor was purchased from Biolin Scientific. Two types of commercial polyamide flatsheet nanofiltration membranes [MICRODYN-NADIR, Germany; molecular weight cutoff (MWCO) of 200 and 800 Da according to the manufacturer’s specification] were used for all experiments.

### Fabrication of the microfluidic filtration device

The high vacuum–compatible microfluidic filtration device was fabricated according to a previously reported method with minor modification (*4*, *38*). In brief, a SiN_x_ film (100 nm in thickness) supported on a silicon frame (window size, 0.5 mm by 0.5 mm) was used as the detection window. The membrane coupon (3.5 mm by 3.5 mm) was carefully immobilized beneath the SiN_x_ film with epoxy resin glue. The active layer of the membrane faced the vacuum side to avoid the rehydration of ions. The SiN_x_ film with the membrane coupon was then sealed on top of a 6.0 mm by 5.2 mm by 1.0 mm (length by width by depth) liquid tank, which was previously manufactured on a poly ether ether ketone (PEEK) block with epoxy resin glue. Two PEEK tubes were also sealed with epoxy resin glue on the left and right sides of the device, with the thick tube (^1^/_16_ inch) serving as the outlet and the thin tube (^1^/_32_ inch) as the inlet. The 10 mM NaX (X = F, Cl, Br) solution was injected through the tube into the liquid tank, which was then sealed with a valve.

### Analysis of hydration distribution by in situ liquid ToF-SIMS

The hydration distributions (*h*_X^−^_) of (H_2_O)_n_X^−^ (X = F, Cl, Br with *n* = 1 to 6) before and after filtration by two polyamide membranes with nominal MWCOs of 200 and 800 Da (denoted as NF 200 and NF 800; fig. S17) were assessed. The feed solutions (i.e., 10 mM NaX; X = F, Cl, Br) were first sealed in the microfluidic filtration device (Fig. 1A) and separated from the vacuum in ToF-SIMS by a SiN_x_ film. The polyamide membrane was fixed between the feed solution and the SiN_x_ film. The microfluidic filtration device was subsequently loaded into the load lock vacuum chamber (<3 × 10^−6^ mbar) and main chamber (1 × 10^−6^ to 2 × 10^−6^ mbar) of the ToF-SIMS V instrument (ION-TOF GmbH, Münster, Germany) to perform in situ liquid SIMS measurements in negative ion mode to obtain the hydration distribution. The conditions of in situ liquid SIMS measurements were modified on the basis of the previously reported work (*4*, *38*). In brief, a 30-keV pulsed Bi_3_^+^ primary ion beam at a frequency of 10 kHz was focused to a diameter of ∼350 nm and scanned on a small circular area 2 μm in diameter on the SiN_x_ film. During SiN_x_ perforation, the pulse width was 162.5 ns, and the current was ~0.27 pA. Once the SiN_x_ film was completely punched through, the pulse width was immediately adjusted to 100 ns for a higher mass resolution, and the corresponding beam current was ~0.15 pA, after which the solution and hydrates were driven across the membrane and reached the detection area driven by an ultrahigh vacuum above the membrane surface (Fig. 1A, inset). The mass spectrum signals of individual hydrates were collected instantly after permeating across the membrane, allowing for acquirement of the hydration distribution under confinement. The control experiment was performed in the microfluidic device with no membrane to analyze the hydration distribution before filtration. All experiments were repeated in triplicate. Details of the data processing are provided in the Supplementary Materials.

### Determination of the energy barriers during ion transport

According to previous work (*16*), the anions and cations transport across the membrane in a decoupled manner; thus, each ion experiences independent energy barriers. On the basis of the TST, we measured the independent apparent transmembrane energy barriers (*E*_a_) of the studied anions by the electric field–driven method (fig. S12C), as shown in an Arrhenius-type equation (Eq. 1)

$$\ln(Gt_{-}T)=\ln(A)-\frac{E_{a}}{R}\frac{1}{T}$$ (1)

where *G* is the overall ion conductance of the single electrolyte solution in our study (i.e., 10 mM NaF, NaCl, and NaBr) through the polyamide nanofiltration membrane. The *t*_−_ is the transport number for the studied anions, which determines the contribution of anion to overall ion conductance and is related to the membrane charge. *A* is the preexponential factor. Accordingly, the energy barriers calculated by the Arrhenius-type equation are the apparent representations (i.e., apparent energy barriers), which reflect contributions from all acting mechanisms during the specific transmembrane process including the influence of transport distance.

The overall ion conductance was measured by current-voltage relation (*I*-*V*) curves using the linear sweep voltammetry (LSV) technique (Interface 1000, Gamry instruments, USA) at different temperatures (i.e., 26°, 30°, 34°, and 38°C). For each measurement, a membrane coupon with an area of 2.01 cm^2^ was assembled between the two chambers of the custom-made electro-driven apparatus (fig. S12C). An Ag/AgCl reference electrode was inserted in each chamber of the apparatus to apply an electric potential across the polyamide membrane. The two chambers were filled with sodium salt solution at the same concentration (10 mM NaX; X = F, Cl, Br). During LSV measurements, the transmembrane potential between the two reference electrodes was scanned from −100 to 100 mV at a scan rate of 2 mV s^−1^ (fig. S18A). The *G* was calculated from the slope of the *I*-*V* curves. Notably, all *I*-*V* curves showed a very good linear relationship (fig. S18A), which indicates that the *G* was constant throughout the measurement. We thus can conclude that from the beginning of the electro-driven process to the end of the test, there was no influence of concentration polarization on the ionic flux. Therefore, the concentration polarization can be ignored in the measurement of transmembrane energy barriers by the electro-driven method.

The transport number (*t*_−_) at the corresponding temperature (i.e., 26°, 30°, 34°, and 38°C) was also analyzed by measuring the membrane potential (ΔΦ_mbr_) based on Eq. 2

$$\text{Δ}{\text{Φ}}_{\mathrm{mbr}}=(1-2t_{-})\frac{RT}{F}\ln\left(\frac{C_{h}}{C_{l}}\right)$$ (2)

where *F* is the Faraday constant (96,485 C mol^−1^). Since polyamide membranes are negatively charged under the experimental pH in our study (pH ≥ 6.0), cations are preferentially transported over anions with *t*_−_<0.5. To determine the membrane potential (ΔΦ_mbr_) at the corresponding temperature, we maintained the electrolyte concentration in one chamber of the electro-driven apparatus to 10 mM (*C*_h_) while gradually reducing the concentration in the other chamber from 10 to 5 and 1 mM (*C*_l_), and the concentration ratios between the two chambers, i.e., $\frac{C_{h}}{C_{l}}$, were 1:1, 2:1, and 10:1, respectively. At each concentration ratio, the abovementioned LSV technique was performed. The horizontal intercept (voltage, mV) of the *I*-*V* curve at different concentration ratios represents the ΔΦ_mbr_. The *t*_−_ was then calculated using Eq. 2 (details in the Supplementary Materials, fig. S18).

The apparent energy of ion partitioning and intrapore diffusion was determined by using the hindered transport theory (*9*, *39*–*42*) in combination with TST (*9*, *16*, *43*). According to previous work (*43*), the apparent activation energy for anion intrapore diffusion (*E*_diffusion_, kcal mol^−1^) can be obtained by substituting the temperature-dependent intrapore diffusion coefficients (*D*_i_, m^2^ s^−1^) into the linearized Arrhenius-type equation (Eq. 3)

$$\ln D_{i}=\ln D_{i0}-\left(\frac{E_{\mathrm{diffusion}}}{R}\cdot \frac{1}{T}\right)$$ (3)

where *D*_i0_ is the preexponential factor (m^2^ s^−1^). According to the hindered transport theory, which operates under the assumption of rigid solutes transporting in cylindrical pores, the intrapore diffusion coefficients (*D*_i_, m^2^ s^−1^) were given as Eq. 4

$$\frac{D_{i}}{D_{W}}=2\int_{0}^{1-\lambda }\left[\frac{f_{\infty }}{f(\beta ,\lambda )}\right]\exp\left(-\frac{E(\beta ,\lambda )}{kT}\right)\beta d\beta$$ (4)

where λ represents the ratio of solute radius (*r*_s_) to pore radius (*r*_p_). *r*_s_ is the effective solute radius being calculated from the Stokes-Einstein relationship (*28*), and *r*_p_ is the effective pore radius being estimated from neutral solute rejection (table S2). *D*_W_ denotes the diffusion coefficients of anions in the bulk solution (table S3), *f*_∞_ is the Stokes friction coefficient for the solute outside the pore, and *f*(β, λ) is the friction coefficient for a solute whose center is at a radial position β and translating parallel to the cylinder axis. *E*(β, λ) is the interaction potential between the solute and the charged pore wall, *k* is Boltzmann’s constant, and *T* is the temperature. Using the centerline approximation, i.e., β = 0, the $\frac{f_{\infty }}{f(\beta ,\lambda )}$ can be known (*40*), and *E*(β, λ) between the solute and charged pore wall can be calculated by

$$E(\beta ,\lambda )=\frac{r_{p}\varepsilon }{4\pi }{\left(\frac{RT}{F}\right)}^{2}\times V$$ (5)

where ε is the solution dielectric constant in the pore, *F* is the Faraday constant, *R* is the ideal gas constant, and *V* is the dimensionless energy factor. For the solid sphere model with constant charge, *V* can be given as

$$V=\frac{\left(\frac{4\pi \tau \lambda^{4}e^{\tau \lambda }S_{0}}{1+\tau \lambda }\right)\sigma_{s}^{2}+\left(\frac{4\pi^{2}\lambda^{2}}{I_{1}(\tau )}\right)\sigma_{s}\sigma_{c}+\left[\frac{\pi^{2}h(\tau \lambda )}{\tau^{2}I_{1}^{2}(\tau )}\right]\sigma_{c}^{2}}{\left[\pi \tau (1+\tau \lambda )e^{-\tau \lambda }-S_{0}h(\tau \lambda )\right]}$$ (6)

where τ is the ratio of pore radius to Debye length, *S*_0_ is the integral factor, *I*_1_(τ) is the modified Bessel function of the first kind, and *h*(τλ) is a function about τ and λ. They can be calculated by

$$\tau =r_{p}{\left[\frac{4\pi F^{2}}{\varepsilon RT}\sum_{i}(z_{i}^{2}C_{i})\right]}^{\frac{1}{2}}$$ (7)

where *z*_i_ and *C*_i_ are the valence and the molar concentration of the electrolyte species *i*.

$$S_{0}=\frac{\pi }{2}{\left(\frac{\pi }{\tau }\right)}^{\frac{1}{2}}e^{-2\tau }\times \left[\tau +\frac{15}{16}-\frac{39}{512\tau }+O\left(\frac{1}{\tau^{2}}\right)\right]$$ (8)

$$h(\tau \lambda )=(1+\tau \lambda )e^{-\tau \lambda }-(1-\tau \lambda )e^{\tau \lambda }$$ (9)

On the basis of the solution-diffusion model, the relationship between partitioning and diffusion can be described as

$$p_{i}=\frac{K_{i}D_{i}}{\delta }$$ (10)

where *p*_i_ is the intrinsic permeability (m s^−1^), *K_i_* is the partitioning coefficient, and δ is the thickness of active layer of the membrane measured by QCM. The intrinsic permeability in an electromigration process can be described as

$$p_{i}=\frac{K_{i}D_{i}}{\delta }=\frac{R}{A_{m}F^{2}C_{i}}Gt_{-}T=\alpha Gt_{-}T$$ (11)

where *A_m_* is the effective membrane area (2.01 cm^2^). Combining Eq. 4 and Eq. 11, *K_i_* can be obtained, and the apparent energy of ion partitioning can thus be calculated by using eq. S8.

The apparent energy of anion diffusion in water can be calculated by the relation between diffusion coefficients of ion diffusion in water (*D*_W_), and the activated jump of hydrates can be described as (*44*)

$$D_{W}=A_{W}\exp\left(\frac{-E_{W}}{RT}\right)$$ (12)

where *E*_W_ is the energy of anion diffusion in water. The *E*_W_ can be calculated by conducting linear regressions on plots of ln(*D*_W_) versus $\frac{1}{T}$. *D*_W_ at different temperatures are given in table S3.

The energy from dehydration can be calculated as follows. First, the partial dehydration of anion can be described as

$${\left(H_{2}O\right)}_{n}X^{-}\text{⇄}{\left(H_{2}O\right)}_{m}X^{-}+(n-m)H_{2}O,\;n>m$$ (13)

According to the definition of reaction enthalpy change and hydration enthalpy (∆*H*_hyd_), the energy of dehydration ($\text{Δ}H_{\mathrm{dehyd}}^{‡}$) was expressed by the difference between hydration enthalpy of hydrates after and before the dehydration

$$\text{Δ}H_{\mathrm{dehyd}}^{‡}=\text{Δ}H_{\mathrm{hyd}}[{\left(H_{2}O\right)}_{m}X^{-}]-\text{Δ}H_{\mathrm{hyd}}[{\left(H_{2}O\right)}_{n}X^{-}]$$ (14)

where ∆*H*_hyd_[(H_2_O)_m_X^−^] and ∆*H*_hyd_[(H_2_O)*_n_*X^−^] are the hydration enthalpy of hydrates after and before dehydration. Since the proportion difference of each hydrate after and before the dehydration follows the *h*_X^−^_ distribution measured by in situ liquid ToF-SIMS, $\text{Δ}H_{\mathrm{dehyd}}^{‡}$ can be calculated by the following equation

$$\text{Δ}H_{\mathrm{dehyd}}^{‡}=\sum_{i}^{n}(\chi_{i}^{\mathrm{after}}-\chi_{i}^{\mathrm{before}})\text{Δ}H_{\mathrm{hyd}}[{\left(H_{2}O\right)}_{i}X^{-}]$$ (15)

where $\chi_{i}^{\mathrm{after}}$ and $\chi_{i}^{\mathrm{before}}$ are the proportion of hydrates with various numbers of strongly bound water molecule (*i* = 1–6) in *h*_X^−^_ distribution after and before filtration by polyamide membrane (Fig. 1) measured using in situ liquid ToF-SIMS. ∆*H*_hyd_[(H_2_O)_i_X^−^] is the hydration enthalpy of corresponding hydrate. ∆*H*_hyd_[(H_2_O)_i_X^−^] was calculated by DFT as shown in fig. S19.

Last, the energy from electrostatic repulsion was calculated on the basis of the assumption that the apparent transmembrane energy barriers (*E*_a_) of anion during transport are mainly from three aspects: (i) dehydration ($\text{Δ}H_{\mathrm{dehyd}}^{‡}$), (ii) ion diffusion in water (*E*_w_), and (iii) electrostatic repulsion (*E*_ele_); *E*_ele_ is given as

$$E_{\mathrm{ele}}=E_{a}-\text{Δ}H_{\mathrm{dehyd}}^{‡}\text{-}E_{w}$$ (16)

### MD simulations and DFT calculations

Atomistic MD simulations were performed in the GROMACS (*45*) (version 2020.6) simulation package using the OPLSAAM force field (*46*–*48*). One hundred polymer chains of four repeat units were randomly inserted into a cubic box of around 5 nm^3^, and a 40-ns MD run under the consant-pressure and constant-temperature (i.e., NPT) ensemble was performed to equilibrate the polymer matrix at the temperature of 600 K. Polymer holes with radii of 3 and 5 Å were generated through packing the equilibrated polymer matrix around the carbon nanotubes (CNTs). All systems with CNT contained 252 chains of polymer and were first equilibrated for 20 ns at 600 K to pack the polymer chains close to the CNT forming holes of exact sizes. Then, the system was annealed within 10 ns to the target temperature of 298 K followed by further equilibration of 10 ns. After removal of the CNT, a ~6.5-nm-long hole of the exact radius was obtained in the polymer matrix. Using such relatively straight pores for simulation may underestimate the contribution of pore tortuosity to energy compared to the real nanofiltration system. However, a previous study indicated that the electrostatic repulsion between the anions and the negatively charged pore wall with ionized carboxyl groups leads anions to move away from the pore wall (*16*), which may reduce the influence of tortuosity on intrapore diffusion in our study. The resulting system was expanded along the *z* axis to 10 nm and filled with more than 8000 water molecules, and the ions were generated through random replacement of water molecules at the concentration of 0.04 M. All systems with F^−^, Cl^−^, and Br^−^ were further equilibrated for another 4 ns with semi-isotropic NPT simulations with the *xy* axis size fixed to equilibrate the water and ions. Last, the PMFs were calculated through umbrella sampling by pulling one of the ions along the *z* axis through the water hole in the polymer matrix with a pulling force constant of 100 kcal mol^−1^ nm^−2^ under the canonical ensemble (i.e., NVT ensemble). Each of the 55 replicas was sampled for 3 to 5 ns while taking the last 2 ns for analysis of the free energy using the g_wham program (*49*). The temperature was controlled by the Nose-Hoover coupling method, and a time step of 2 fs was used for the integrations. A cutoff length of 1.2 nm was implemented for the nonbonded interactions, and the particle mesh Ewald method (*50*) with a Fourier spacing of 0.1 nm was applied for the long-range electrostatic interactions. All covalent bonds with hydrogen atoms were constrained using the LINCS algorithm (*51*).

The structures of various (H_2_O)_n_X^−^ were investigated by ab initio DFT. The M06-2X is one of the most suitable functions for the study of noncovalent interactions (*52*), which includes halide ion-water interactions. For basis sets used for geometry optimization and energy calculation of systems containing weak interactions, the def2-TZVP level has ideal accuracy and suitable computing resource requirements. Therefore, the M06-2X/def2-TZVP method is a popular choice for geometry optimization and energy analysis of hydrated ions (*53*). Thus, the most probable geometries of all (H_2_O)_n_X^−^ (*n* = 1 to 6; X = F, Cl, and Br) were optimized at the M06-2X/def2-TZVP level using the density functional method (*52*). Then, to improve the calculation accuracy, ma-def2-TZVP basis set was used to calculate the total electron energy for all molecules. All these calculations were performed with the Gaussian 16 software package (*54*). The most probable geometries of (H_2_O)_n_X^−^ were searched by using the Boltzmann distribution law (*55*) based on relative energy. Considering the huge computational effort for energy calculations of all configurations, we obtained the relative binding energies (∆*E_j_*) from previous studies (*31*, *32*). According to the Boltzmann distribution law, the proportion of *j* configuration (*P_j_*) can be given as

$$P_{j}=\frac{n_{j}}{\sum n_{j}}=\frac{e^{-\text{Δ}E_{j}/RT}}{\sum e^{-\text{Δ}E_{j}/RT}}=\frac{Q_{j(\mathrm{Relat})}}{Q_{\left(\mathrm{Relat}\right)}}$$ (17)

where *n_j_* is the molecule number of hydrates with *j* configuration, *R* is the gas constant, *T* is the absolute temperature, *Q*_*j*(Relat)_ is the distribution function of hydrates with *j* configuration, and the subscript “Relat” represents that the *Q*_*j*(Relat)_ was calculated from relative binding energies. The *P_j_* of each configuration of (H_2_O)_n_X^−^ is shown in table S4.

### Investigation of ion transport and selectivity under different driving forces

According to the Donnan-exclusion mechanisms, the performance of a nanofiltration membrane is normally dictated by the co-ions with identical charge to the membrane surface (*56*), which are anions in our study, because polyamide membranes are negatively charged under experimental pH (≥6.0). We conducted sets of filtration experiments to evaluate the anion transport and selectivity of nanoporous polyamide membranes under different driving forces. Two polyamide nanofiltration membranes (fig. S17) with nominal MWCO of 200 and 800 Da (denoted as NF 200 and NF 800) were selected to investigate the role of steric effects. Membrane surface charges were controlled by varying the ionization of the carboxyl groups under different solution pH for evaluating the role of electrostatic repulsion. Before the experiment, all membrane coupons were agitated in 25% 2-propanol solution for 0.5 hours using a rotating shaker. Then, the membrane coupons were carefully rinsed with DI water and kept in DI water overnight to remove impurities.

#### Ion transport and selectivity in nanofiltration

A custom-made filtration system with a flat sheet nanofiltration membrane cell driven by pressure, i.e., nanofiltration, was used for ion transport and selectivity tests (fig. S12A). The nanofiltration system was operated in cross-flow mode. The effective area of the membrane sheet was 20.6 cm^2^. Both retentate and permeate were recirculated to the feed tank. The membrane was compacted by DI water under a pressure of ~8.0 bar until the water flux was steady. The pressure was then reduced to ~6 bar, followed by the addition of salt solution (NaF, NaCl, and NaBr) to obtain a 4 mM feed solution to start the experiments. Unless otherwise indicated, the water temperature was maintained at a constant temperature of 20°C, and the cross-flow velocity was 19.8 cm s^−1^. On the basis of the solution diffusion model (*14*), intrinsic permeability of the different ions (*p*_i_, m s^−1^) was calculated from the measured flux and concentration difference across the membrane

$$p_{i}=\frac{J_{i}}{C_{0}-C_{p}}$$ (18)

where *J_i_* is the ion flux (mol m^−2^ s^−1^) and *C*_0_ and *C*_p_ are the concentration of the feed and permeate solution (mM), respectively. *C*_0_ and *C*_p_ were analyzed by ion chromatography (ICS-2000, Dionex, USA). The selectivity ratio (*SR*_X^−^/F^−^_) was defined as the ratio of the intrinsic permeability between X^−^ (i.e., F^−^, Cl^−^, and Br^−^) and F^−^.

#### Ion transport and selectivity in diffusion

Ion transport experiments driven by concentration gradient, i.e., diffusion, were conducted in a custom-made diffusion cell (fig. S12B). Two diffusion chambers were separated by a membrane coupon (with an effective area of 2.01 cm^2^). The left-side chamber was filled with 20 ml of concentrated salt solution (*C*_0_ = 0.1 M), while the other side was filled with isopycnic DI water as the receiver. All diffusion experiments were constantly stirred with a magnetic stirrer to avoid concentration polarization. Samples from the receiving chamber were collected after 3 hours of diffusion. The concentration of anions in the receiving solution (*C*_p_) was determined by ion chromatography (ICS-2000, Dionex, USA) to calculate the intrinsic permeabilities of the different ions (Eq. 18).

#### Ion transport and selectivity in electromigration

Ion transport in an electro-driven process, i.e., electromigration, was studied by *I*-*V* curves using the LSV technique (Interface 1000, Gamry instruments, USA). Ionic conductance (*G*) was measured using the same two-chamber apparatus as mentioned in the diffusion experiment (fig. S12C). An Ag/AgCl reference electrode was inserted in each chamber to apply an electric potential across the polyamide membrane. The two chambers were filled with sodium salt solution at the same concentration (0.1 M NaX; X = F, Cl, Br). The transmembrane potential between the two reference electrodes was scanned from −100 to 100 mV at a scan rate of 2 mV·s^−1^. The intrinsic permeability in an electromigration process can be described as (*16*)

$$p_{i}=\frac{DK}{\delta }=\frac{R}{A_{m}F^{2}C}Gt_{-}T=\alpha Gt_{-}T$$ (19)

where *D* is the diffusion coefficient, *K* is the partition coefficient, δ is the thickness of active layer of membrane, *R* is the gas constant, *A_m_* is the effective membrane area (2.01 cm^2^), *F* is the Faraday constant, *C* is the electrolyte molar concentration in the bulk solution, *t*_−_ is the transport number, *T* is the absolute temperature, and α is a temperature-independent parameter. The δ can be determined by QCM (details in the Supplementary Materials, note S8).

The pH of the electrolyte solutions was adjusted using 0.1 M NaOH and HCl solutions. Each test was repeated at least three times to obtain the average values under different conditions.

## Supplementary Materials

### This PDF file includes:

Supplementary Text Figs. S1 to S24 Tables S1 to S5 References
