# Supplementary material for: Dehydration-enhanced ion-pore interactions dominate anion transport and selectivity in nanochannels
Source: Sci Adv. 2023 Jul 7;9(27):eadf8412. doi: 10.1126/sciadv.adf8412 (PMC10328398; doi:10.1126/sciadv.adf8412)
Supplement: Supplementary file 1 — Supplementary Text Figs. S1 to S24 Tables S1 to S5 References [file sciadv.adf8412_sm.pdf]

Science Advances
AAAS

Supplementary Materials for

Dehydration-enhanced ion-pore interactions dominate anion transport and selectivity in nanochannels

Chenghai Lu *et al.*

Corresponding author: Chengzhi Hu, czhu@rcees.ac.cn; Yanyan Zhang, zhangyy0816@iccas.ac.cn; Jiuhui Qu, jhqu@rcees.ac.cn

Sci. Adv. **9**, eadf8412 (2023)
DOI: 10.1126/sciadv.adf8412

This PDF file includes:

Supplementary Text
Figs. S1 to S24
Tables S1 to S5
References

Supplementary Text

Note. S1. Device and membrane characterization

Membrane pore size distributions were estimated using inert solutes and the pore transport model as our previous work reported (*4, 57*). The concentration of neutral organic molecules used here was determined by a total organic carbon (TOC) analyzer (TOC-VCPH, Shimadzu, Japan). The chemical properties of the membranes were analyzed by performing attenuated total reflectance Fourier transform infrared (ATR-FTIR, Nicolet 8700, Thermo Fisher Scientific, USA). The ionized carboxyl density within the polyamide membrane was investigated by a silver elution method (*58*). Eluted silver ions were quantified using inductively coupled plasma mass spectrometry (ICP-MS,7500a, Agilent Technologies Inc., USA). The zeta potential of membrane surface was evaluated by an electrokinetic analyzer for solid surface analysis (SurPASS 3, Anton Paar, Austria).

Note. S2. Detailed discussion of *in situ* liquid ToF-SIMS data processing

We have described in detail the principle of the microfluidic filtration platform for *in situ* ToF-SIMS analysis in our previous work (*4*). The microfluidic filtration platform was illustrated in Fig. 1A. In brief, a polyamide membrane coupon was sealed between the feed solution (10mM NaX, X=F, Cl, Br) and SiN_x film. Once the SiN_x film was completely punched through by a pulsed Bi_3^+ primary ion beam, water and ion were driven across the membrane by pulling an ultrahigh vacuum at the membrane surface to reach the detection area. The hydrates in NaX solution were consequently exposed to the Bi_3^+ primary ion beam and were immediately ionized to secondary ions, forming the instantaneous mass spectrum of interested hydrates. As shown in dynamic depth profiling with ToF-SIMS (Fig. S20A), the perforation of the SiN_x film occurred at roughly 445 s, which was marked by a notable reduction in the Si^- signals and a simultaneous increase in the $(H_2O)Cl^-$ signals from the NaX solution. We selected the mass spectrum data of various hydrates at the moment of SiN_x perforation from the detection area (see reconstructed 2D chemical images, Fig. S20B). Through these steps, the mass spectrum of the interested hydrates in the bulk solution (i.e., before filtration) and permeate (i.e., after filtration) were obtained (Fig. S20C). The mass spectrum peaks of various hydrates were assigned by their mass-to-charge ratio (m/z). The collected mass spectrum peak intensities of individual hydrate $[I_{(\mathrm{H_2O})_n\mathrm{X}^-}]$ were normalized to the total mass spectrum intensities of all hydrated ion $[\sum I_{(\mathrm{H_2O})_n\mathrm{X}^-}]$ to obtain the ion hydration distribution (h_{X^-}), i.e.,

$$\chi_n = \frac{I_{(H_2O)_nX^-}}{\sum_{n=1}^{n=6} I_{(H_2O)_nX^-}}, n = 1-6 \quad \text{(S1)}$$

To better describe the hydration distribution, the weighted average of each hydration distribution was calculated for mathematical comparison, i.e.,

$$h_{avg} = \frac{\sum n\chi_n}{\sum \chi_n} = \sum n\chi_n, n = 1-6 \quad \text{(S2)}$$

Notably, although the *in situ liquid* ToF-SIMS has been demonstrated to be a soft ionization technique for the detection of solvation structures and weak ion-water interactions, the hydrated water of the ion is not completely prevented from being damaged by the Bi_3^+ beam bombardment during the *liquid* ToF-SIMS performance. In addition, the secondary ion yields in SIMS analysis are influenced by many factors. Thus, the experimentally obtained hydration number, e.g., h_{avg} may have deviations from the real hydration numbers of ions in the liquid phase. However, this would not affect the accuracy of the interrelationship between the h_{X^-} of various hydrates in the same test system. The differences in the numbers of the detected bound waters for the different ions or experimental conditions are informative and representative of a real difference in the liquid phase.

Note. S3. Discussion on dehydration vs rearrangement of hydration shell

In our study, we defined the dehydration vs. rearrangement in two aspects: (i) the change in hydration numbers (HNs) and (ii) the distance between anion and water molecule. For hydrates entering the pore channel with a reduced strongly-bound HNs, this can clearly be described as "dehydration" (*8, 20, 27*). In the case of hydrates that enter pores smaller than their hydrated size (i.e., $\mu_p < r_{H-X^-}$) but maintained the same strongly-bound HNs as in the bulk, they should compress or deform the hydration layer for fitting into the pore, which can be described as "rearrangement" (*8, 20, 27*). The strongly-bound HNs can be determined by liquid ToF-SIMS. While in the MD simulations, the HNs are defined as the number of hydrated water molecules within the most probable interaction distance between X^- and the water molecule. That is, if the distance between the hydrated water and the ion exceeds this value, the hydrated water will no longer interact with the ion, i.e., dehydration occurs. When the hydration shell of hydrates is deformed or compressed but the hydrated water remains within this range, the hydration layer may have been rearranged.

For strongly hydrated ions, their higher hydration energy and more negative hydration entropy contribute to a larger and rigid/compact hydration shell (*12, 20, 59*). These larger strongly hydrated ions, are thus less readily to compress and deform the hydration layer than the weakly hydrated ions, or even if their hydration layer has been rearranged, they still cannot fit into the pore and must

strip part of hydration shell to enter the pore due to their larger size. In contrast, for ions with lower hydration energy, i.e., weakly hydrated ions, their hydration shell is smaller and softer (*12, 59*). The smaller size facilitates the transport through the nanochannel only by the rearrangement of hydration layer, indicating that the rearrangement of hydrated shells is energetically more favorable than dehydration (*12, 27*).

In our study, the pore sizes of the sterically limited membrane and the estimated hydrodynamic radii of hydrates (r_{H-X^-}) follow the order: r_{H-F^-} (0.352 nm) > r_{H-Cl^-} (0.332 nm) > r_{H-Br^-} (0.330 nm) > $\mu_{p,\ NF\ 200}$ (0.30 nm). These three ions should dehydrate or rearrange their hydration shell according to the aforementioned theory. We did observe a decrease in the h_{X^-}, i.e., dehydration of $(H_2O)_nF^-$ and $(H_2O)_nCl^-$ (Fig. 1B-1C and Fig. S3A-S3B), but the h_{Br^-} did not decrease during transport in sterically limited membrane (Fig. 1D and Fig. S3C). Therefore, we deduce that the $(H_2O)_nBr^-$ are more likely to rearrange the hydration layer for entering the sterically limited membrane. Collectively, we can infer the dehydration or hydrated shell-rearrangement of ions according to the size relationship between ions and pores and the observed strongly-bound HNs during the transport.

Note. S4. Surface vs. internal structure of $(H_2O)_nX^-$

For hydrated ions, there are two types of geometries to describe the structure of solvated ions. A surface structure (*S*) will correspond to the ion being located on the top/surface of the water molecules, which are bound together by water-water hydrogen bonds (HBs) (*29, 30*). $(H_2O)_4Cl^-$ and $(H_2O)_4Br^-$ are good examples of surface structure (Fig. S4). In an internal structure (*I*), the bound water molecules surround the ions creating a cage for the ion (*29, 30*). The geometry of hydrated ions is determined by the balance between the water-water interaction and ion-water interaction (*30, 31, 60*). The *S*-structures are characterized by the stronger water-water interaction, which forms a water cluster on one side of the ion. In contrast, for the *I*-structure, the stronger ion-water interaction renders the arrangement of water molecules around the central ion.

Thus, we calculated the geometric parameters of $(H_2O)_nX^-$ to analyze the water-water interaction and ion-water interaction (Table. S5). We note that the water-water HBs numbers of $(H_2O)_nF^-$ were less than that of $(H_2O)_nCl^-$ and $(H_2O)_nBr^-$, the distances between X^- and water molecules (r_{X-H}) of $(H_2O)_nF^-$ were also shorter than those of $(H_2O)_nCl^-$ and $(H_2O)_nBr^-$. This indicates that the F^--water interaction is stronger than Cl^--water and Br^--water interaction. To further investigate the balance between the water-water interaction and ion-water interaction of each $(H_2O)_nX^-$, we compare the O-H bond length of water molecules interacting with X^-, r_{O-H_X}, and

those interacting with neighboring water, $r_{\mathrm{O-H_w}}$, respectively. For $(H_2O)_nF^-$ (n=1-6), the $r_{\mathrm{O-H_F}}$ of all hydrates were longer than the $r_{\mathrm{O-H_w}}$, showing stronger F^--water interaction than water-water interaction in $(H_2O)_nF^-$ (Table. S5). Conversely, for $(H_2O)_nCl^-$ and $(H_2O)_nBr^-$, the $r_{\mathrm{O-H_{Cl}}}$ and $r_{\mathrm{O-H_{Br}}}$ were generally shorter than those of $r_{\mathrm{O-H_w}}$ except for the hydrates with less bound water (n=1-2, Table. S5). This indicates that the water-water interaction is stronger that the X-water interaction in $(H_2O)_nCl^-$ and $(H_2O)_nBr^-$ (n=1-4). Combining these geometric parameters and observed configurations of $(H_2O)_nX^-$, it can be inferred that the $(H_2O)_nCl^-$ and $(H_2O)_nBr^-$ favor surface structures, while $(H_2O)_nF^-$ favors semi-surface structure due to the stronger F-water interaction (*31, 32*). Since the water molecules in the *I* structure are more evenly distributed around the anion, the *I*-structure is relatively more sensitive to the confinement of nanochannel than the *S*-structure where the water molecules are concentrated on one side of the anion.

Note. S5. Estimation of membrane pore size distributions

The pore size distribution (PSD) curves of all membranes were characterized by means of the solute transport method as reported elsewhere (*4, 57*). The PSD curve of a polyamide membrane is given by the following probability density function:

$$\frac{dR_{\mathrm{T}}(r_{\mathrm{p}})}{dr_{\mathrm{p}}} = \frac{1}{r_{\mathrm{p}}\ln\sigma_{\mathrm{p}}\sqrt{2\pi}} \cdot \exp\left[-\frac{(\ln r_{\mathrm{p}} - \ln\mu_{\mathrm{p}})^2}{2(\ln\sigma_{\mathrm{p}})^2}\right] \tag{S3}$$

where R_{T} is the solute rejection, r_{p} is the effective pore radius of the membrane. By ignoring the effects of steric hindrance and solute-pore hydrodynamic interactions on R_{T}, the pore size can be described by solute radius. Accordingly, the mean effective pore radius (μ_{p}) is given as the solute radius (r_{s}) at R_{T} = 50%. The geometric standard deviation (σ_{p}) of the effective pore raidus is defined as the ratio of r_{s} at R_{T} = 84.13% over than that at R_{T} = 50%.

R_{T} is linearly related to r_{s} in the log-normal probability coordinate system:

$$f(R_{\mathrm{T}}) = a + b(\ln r_{\mathrm{s}}) \tag{S4}$$

The effective solute rejection R_{T} (%) was determined with a total organic carbon (TOC) analyzer.

A set of 200 ppm neutral organic solutions containing ethylene glycol, diethylene glycol, polyethylene glycol (PEG-200, PEG-400, PEG-600 and PEG-800) were used to analyze the relationship between R_{T} and r_{s}. The r_{s} (nm) can be described by the molecular weight (MW, Da) as following:

$$\log \mathrm{r_s} = \text{-}1.5575 + 0.4911\log\mathrm{MW} \tag{S5}$$

The PSD curves of the membranes used in our study were calculated according to Eq. S3, as shown in Fig. S1 and Fig. S21.

Note. S6. Quantification of ionized carboxyl density in polyamide membrane.

The ionized carboxyl density within the polyamide membrane was investigated by a silver elution method reported elsewhere (*58, 61*). We prepared 40 μM silver nitrate ($AgNO_3$) and 1 μM $AgNO_3$ for the silver binding and surface washing steps, respectively. 0.1 M NaOH and HCl were used to adjust the pH of the binding and washing solution. The membrane coupons were wetted in DI water for 30 minutes and then cut into 2 cm × 1 cm (length × width) for use. The polyester fabric backings were physically removed to avoid additional Ag^+ binding by backings, after which membranes were immersed in 10 mL of binding solution (40 μM $AgNO_3$) for 10 minutes each time (repeated twice). After the binding step, the membranes were carefully transferred to 10 mL of washing solution (1 μM $AgNO_3$) for 7 minutes each time (repeated 4 times) at the same pH to rinse off the unbound Ag^+. Membranes were subsequently blotted dry and immediately immersed in 5 mL of 1% HNO_3 for 30 minutes to protonate the carboxyl group and elute bound Ag^+.

Eluted Ag^+ was further quantified by inductively coupled plasma mass spectrometry (ICP-MS) and converted to ionized carboxyl density follow the assumption that each eluted Ag^+ was bound to one ionized carboxyl group (i.e., 1:1 binding between Ag^+ and R-COO^-). According to previous work (*58*), only partial surface carboxyl groups are ionized in polyamide membranes when the pH is 6.0, therefore the carboxyl density at pH 6.0 is given by the areal R-COO^- density as follows:

$$[R-COO^-]_{\mathrm{areal}} = \frac{C_{\mathrm{Ag^+}} \times V_{\mathrm{Ag^+}}}{A_{\mathrm{m}}} \times N_{\mathrm{A}} \qquad \text{(S6)}$$

where $[R-COO^-]_{\mathrm{areal}}$ is the areal ionized carboxyl density (sites nm^{-2}), $C_{\mathrm{Ag^+}}$ is the measured molar concentration of elute Ag^+ at pH 6.0, $V_{\mathrm{Ag^+}}$ is the elution volume (5 mL), N_{A} is Avogadro's number and A_{m} is the projected surface area of the polyamide membrane (2 cm^2).

At pH 10.5, all carboxyl groups, including inside of the membrane, are completely ionized. The carboxyl group density is thus given by the volumetric R-COO^- density as follows:

$$[R-COO^-]_{\mathrm{volumetric}} = \frac{C_{\mathrm{Ag^+}} \times V_{\mathrm{Ag^+}}}{A_{\mathrm{m}} \times \delta} \times N_{\mathrm{A}} \qquad \text{(S7)}$$

where $[R-COO^-]_{\mathrm{volumetric}}$ is the volumetric ionized carboxyl density (sites nm^{-3}), $C_{\mathrm{Ag^+}}$ is the measured molar concentration of elute Ag^+ at pH 10.5, δ is the measured membrane thickness by QCM (see Note. S8).

Note. S7. The ionization behavior of carboxyl in polyamide membranes

The ionization behavior of carboxyl in NF 200 and NF 800 was shown in Fig. S13. At pH 6.0, only part of carboxyl groups in membrane surface were ionized (*58*). It was shown that $[R-COO^{-}]_{\text{areal}}$(NF 200) < $[R-COO^{-}]_{\text{areal}}$(NF 800), which is consistent with the results of Zeta potential (Fig. 4D). When pH was increased to 10.5, all carboxyl groups, including surface and inside of the active layer in polyamide membrane were completely ionized (*58*). We find similar value of Zeta potential between the completely ionized surface of NF 200 and NF 800 (Fig. 4D). At the same time, $[R-COO^{-}]_{\text{volumetric}}$ of the two membranes were close (Fig. S13), indicating that the content of ionized carboxyl groups inside the active layer was mainly determined by the membrane thickness. With the thicker active layer, NF 200 thus has similar negatively charged surface but longer negatively charged channels compared to NF 800 at pH 10.5. However, our research shows that interactions between negatively ionogenic groups inside the nanochannels in polyamide membrane, i.e., intrapore diffusion were not the rate-limiting step for anion transport (*4, 9, 13, 18, 62*). Therefore, the different transport behavior of anions was mainly related to the electrostatic interactions near the membrane pore entrance and steric effect.

Note. S8. Measurement of apparent energy during ion partitioning into nanochannels using QCM

According to the solution-diffusion theory, ion permeation through the active layer of nanofiltration membrane can be divided into two processes: ion partitioning into the active layer and diffusion through the active layer. Partitioning covers the interactions between ions and membrane entrance to enter inside of membrane. The apparent energy of salts to partition ($E_{\text{p, salt}}$) can be described by an Arrhenius-type equation (*16*):

$$\ln(K) = \ln(A_{\text{K}}) - \frac{E_{\text{p,salt}}}{R}\frac{1}{T} \quad \text{(S8)}$$

where K is the partition coefficient, A_{K} represents the pre-exponential factor in partition. Quartz crystal microbalance (QCM, Biolin Scientific) analysis was used to measure the partition coefficient (K) of different NaX solution (0.5 M NaF, NaCl and NaBr) at corresponding temperature (26, 30, 34 and 38 ºC) as reported elsewhere (*16, 63*). All QCM experiments were carried out under ambient pH (pH 6.0).

Preparation of QCM sample. As is shown in Fig. S22, to immobilize the active layer of polyamide membrane on the QCM sensor, the fabric backing of membrane coupon was firstly peeled off leaving the polysulfone and polyamide active layer. The isolated two-layered membrane was placed

and pressed gently on a QCM sensor to avoid wrinkling (polysulfone layer facing air). A few drops of *N*, *N*-Dimethylformamide (DMF) were then added on the isolated two-layered membrane to dissolved the polysulfone layer. Repeat this step to ensure that the polysulfone layer was completely dissolved, after which the fixed polyamide active layer was carefully rinsed in clean DMF and DI water. Dry samples at room temperature.

Determination of salt partition coefficient (*K*) in polyamide membrane. To determine the salt partition coefficient, the volume of active layer is needed. We first measured the areal mass of the active layer coated on QCM sensor (m_a) based on the Sauerbrey relationship (*16*):

$$m_\mathrm{a} = \frac{C}{n} \times \Delta f_\mathrm{a} \tag{S9}$$

where n is the overtone number (n = 3 in this study), C is the crystal constant (17.7 ng Hz^{-1} cm^{-2}) and Δf_a is the resonance frequency change between the coated and uncoated membrane QCM sensor tested in air. The thickness of the active layer was then calculated by:

$$\delta = \frac{m_a}{\rho} \tag{S10}$$

The volumetric mass density (ρ) of the polyamide membrane is 1.20 g cm^{-3} in this study according to the manufacturer. Thus, the volume of active layer (V_a) can be calculated as:

$$V_\mathrm{a} = \delta \times A_\mathrm{m} \tag{S11}$$

where A_m is the membrane area isolated on each sensor (A_m=0.78 cm^{-2} in our study). Hence, the molar concentration (C_a) of salts in the active layer of polyamide membrane by partitioning can be determined as:

$$C_\mathrm{a} = \frac{m_\mathrm{p,a} \times A_\mathrm{m}}{MW} \times \frac{1}{V_\mathrm{a}} \tag{S12}$$

where $m_\mathrm{p,a}$ is the areal mass of salts entering the active layer of polyamide membrane by partitioning, and *MW* is the molecular weight of the corresponding salts.

We measured $m_\mathrm{p,a}$ using a QCM (Fig. S23A) based on the Sauerbrey relationship (Eq. S9). Before the salt partitioning test, the coated QCM sensor was first balanced using DI water to establish a stable resonance frequency baseline (Fig. S23B and S23C). Then, the salt solutions (0.5M NaX, X=F, Cl, Br) were pumped into the sensor (pump speed was 0.1 ml min^{-1}) for salt solutions partitioning process under different temperature (26, 30, 34, 38 ºC) until reaching a new stabilized frequency reading (Fig. S23B and S23C). To eliminate the influence of physical and chemical properties of solution on the resonance frequency (e.g., viscosity), we conducted a control test with an uncoated QCM sensor, which followed the same protocol as the coated sensor. Thus,

the change in resonance frequency resulting from the salt partitioning into active layer of polyamide membrane ($\Delta f_{\mathrm{p,a}}$) can be calculated as

$$\Delta f_{\mathrm{p,a}} = \Delta f_{\mathrm{p}} - \Delta f_{\mathrm{c}} \quad \text{(S13)}$$

where Δf_{p} and Δf_{c} are the resonance frequency change for coated sensor and uncoated sensor (control test), respectively. Thus, $m_{\mathrm{p,a}}$ can be calculated using Eq. S9 and K can be accordingly determined by its definition:

$$K = \frac{c_{\mathrm{a}}}{c_{\mathrm{b}}} \quad \text{(S14)}$$

where c_{b} is the salt concentrations in the bulk solution (0.5 M).

Note S9. Transition-state theory (TST) calculation

Measurement of the entropic and enthalpic component of apparent transmembrane energy. The entropic ($-T\Delta S^{\ddagger}$) and enthalpic ($\Delta H^{\ddagger}$) component of anions transport through the polyamide membrane were calculated according to the Eyring's TST (*9, 14, 64*), which assumed that the traversing hydrates make point-to-point jumps through the membrane governed by a rate constant:

$$p_{\mathrm{i}} = \frac{\lambda^2}{\delta}\left(\frac{kT}{h}\right)\exp\left(\frac{-\Delta H^{\ddagger}}{RT}\right)\exp\left(\frac{\Delta S^{\ddagger}}{R}\right) \quad \text{(S15)}$$

where p_{i} is the intrinsic permeability, λ is the distance between equilibrium positions in the membrane, δ is the membrane thickness, k is the Boltzmann constant, h is Planck's constant, $\Delta H^{\ddagger}$ is the enthalpic component, R is the gas constant and $\Delta S^{\ddagger}$ is the entropy change of hydrates during transport. We measured p_{i} at different temperature (i.e., 26, 30, 34 and 38 ºC). The entropic and enthalpic component thus can be calculated by:

$$\ln\left(\frac{p_i \delta h}{\lambda^2 kT}\right) = \frac{\Delta S^{\ddagger}}{R} - \frac{\Delta H^{\ddagger}}{RT} \quad \text{(S16)}$$

The value of λ was taken as an average of 0.5 nm according to Sharma and Chellam's study of electrolyte permeation across polyamide membranes (*65*). The membrane thickness (δ) was measured by aforementioned QCM method.

Note S10. Calculation of effective charges of $(H_2O)_nX^-$

The effective charges ($C_{\mathrm{effective}}$) of $(H_2O)_nX^-$ were calculated according to the formula (*35*):

$$C_{\mathrm{effective}} = \frac{ne}{V_{\mathrm{hyd}}} \quad \text{(S17)}$$

where n is the ion charge (-1), e represents the electron charge (1.60×10^{-19} C) and V_{hyd} is the molecule volume of corresponding hydrates $(H_2O)_nX^-$, which is calculated by DFT (Fig. S9).

Note. S11. Concentration polarization and real intrinsic permeability

To determine the real intrinsic permeability after considering concentration polarization, we calculated the mass transfer coefficient at the boundary layer on the membrane according to the film theory (*66, 67*). The real anion concentration on membrane surface after considering concentration polarization was calculated according to the equation:

$$\frac{R_{\mathrm{real}}/(1-R_{\mathrm{real}})}{R_{\mathrm{obs}}/(1-R_{\mathrm{obs}})} = \frac{C_{\mathrm{m}}-C_{\mathrm{p}}}{C_0-C_{\mathrm{p}}} = \exp\left(\frac{J_{\mathrm{V}}}{k}\right) \quad \text{(S18)}$$

where R_{real} is the real rejection after considering concentration polarization, R_{obs} is the observed rejection, C_{m} is the real anion concentration on membrane surface after considering concentration polarization (mol L^{-1}), C_0 and C_{p} are the concentration of the feed and permeate solution (mol L^{-1}) respectively, J_{V} is the volume flux of permeate (L m^{-2} h^{-1}) and k is the mass transfer coefficient (m s^{-1}).

The k can be calculated as:

$$k = \frac{S_h \times D}{d_h} \quad \text{(S19)}$$

where D is the solute diffusion coefficient (Table. S3), d_h is the hydraulic diameter of cross-flow nanofiltration membrane cell (0.00434 m). The Sherwood number S_h can be expressed as the following equation in our nanofiltration system:

$$S_h = 1.85\left(R_e \times S_c \times \frac{d_h}{L}\right)^{0.33} \quad \text{(S20)}$$

where R_e is the Reynolds number, S_c is the Schmidt number and L is the characteristic length (0.0452 m).

The real intrinsic permeability of anions in the nanofiltration at the corresponding temperature (20 °C) can be calculated based on Eq. S18 and Eq. 18 in the manuscript.

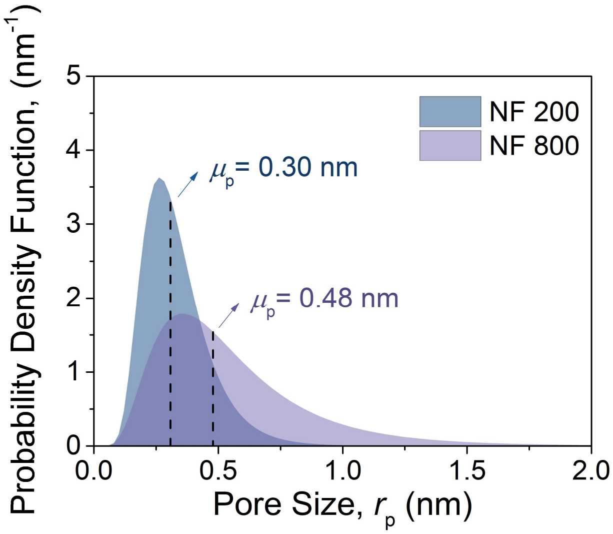

Fig. S1. Pore size distributions (PSDs) of the polyamide membranes. PSDs are represented as probability density function curves. The pore size of the membranes can be divided in two types: (i) sterically limited, where pores are smaller than the size of traversing solvated anions, i.e., NF 200 with mean effective radii (μ_p) of 0.30 nm, and (ii) non-sterically limited, where pores are larger than the size of traversing solvated anions, i.e., NF 800 with μ_p of 0.48 nm.

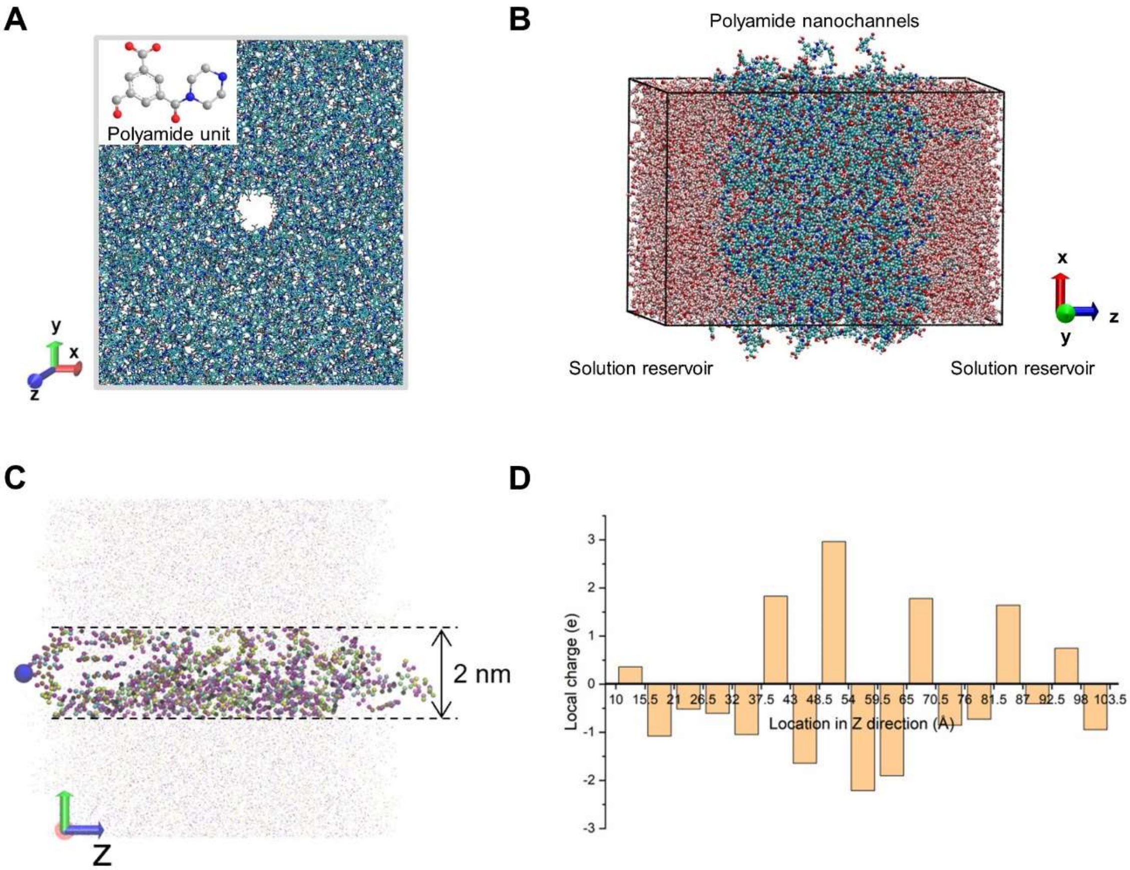

Fig. S2. MD simulation platform of the polyamide nanochannels. (A) Simulated bulk polyamide with nanochannel, where carbon, nitrogen and oxygen atoms are shown as cyan, blue and red, respectively. The highly cross-linked and porous polymeric membrane was simplified to a nanochannel composed of piperazine amide. The inset represents the repeating polypiperazine amide unit for constructing the polymeric nanochannel. Carbon, nitrogen, and oxygen atoms are depicted as gray, blue and red, respectively. **(B)** The cross-sectional illustration of the simulation platform. The water-filled reservoirs with NaX solution (X=F, Cl and Br) were connected by a polyamide nanochannel to form the simulation platform. **(C)** The radial range of ~2 nm to analyze the charge distribution in the center of the R≈0.3nm nanochannel along Z-direction. **(D)** The charge distribution of the $R\approx0.3$nm nanochannel along Z-direction.

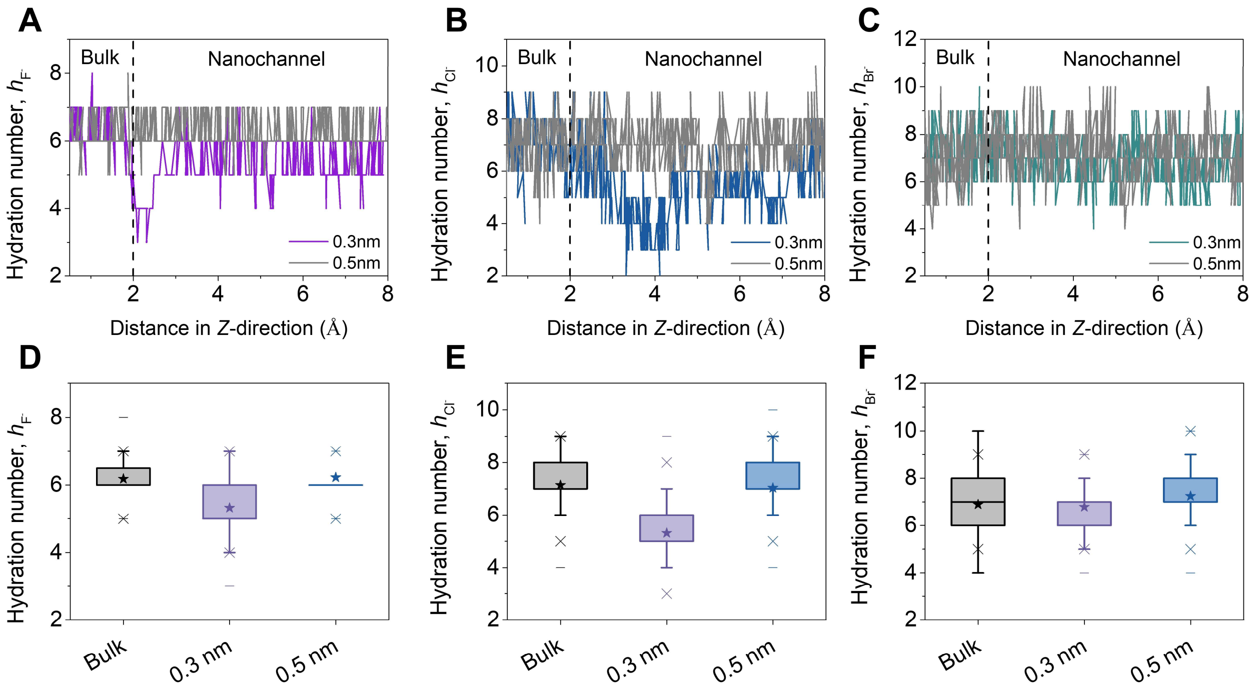

Fig. S3. MD simulation for verification of the hydration change during $(H_2O)_nX^-$ transport through polyamide nanochannel. (A)-(C) The simulated hydration number (h_{X^-}) as a function of distance in the Z-direction from bulk solution into the polyamide nanochannels for $(H_2O)_nF^-$, $(H_2O)_nCl^-$ and $(H_2O)_nBr^-$, respectively. **(D)-(F)** Box chart showing h_{X^-} change of $(H_2O)_nF^-$, $(H_2O)_nCl^-$ and $(H_2O)_nBr^-$ when transport from bulk into the polyamide nanochannels with different size. The h_{X^-} in different position of Z-direction during ion transport in the bulk, 0.3 nm nanochannels and 0.5 nm nanochannels were collected and counted to show as a box chart. It is of note that the h_{X^-} in bulk from MD simulation of 6 to 8 were larger than those measured by ToF-SIMS. This could be due to a certain degree of hydrated water loss caused by Bi_3^+ beam bombardment during liquid ToF-SIMS measurement. We also found that the h_{X^-} measured by ToF-SIMS were in good agreement with the h_{X^-} from theoretical extractions based on bulk properties (*68-71*), which range from 1.2 to 4.9. The h_{X^-} from bulk properties refer to those solvent molecules that are bound strongly to the ion and move along with it in the solution. Hence, the h_{X^-} determined by liquid ToF-SIMS can be describe as strongly-bound water number. Notably, within the same liquid ToF-SIMS test system, the differences in the numbers of the detected bound waters for the different ions or experimental conditions are still informative and representative of a real difference in liquid phase.

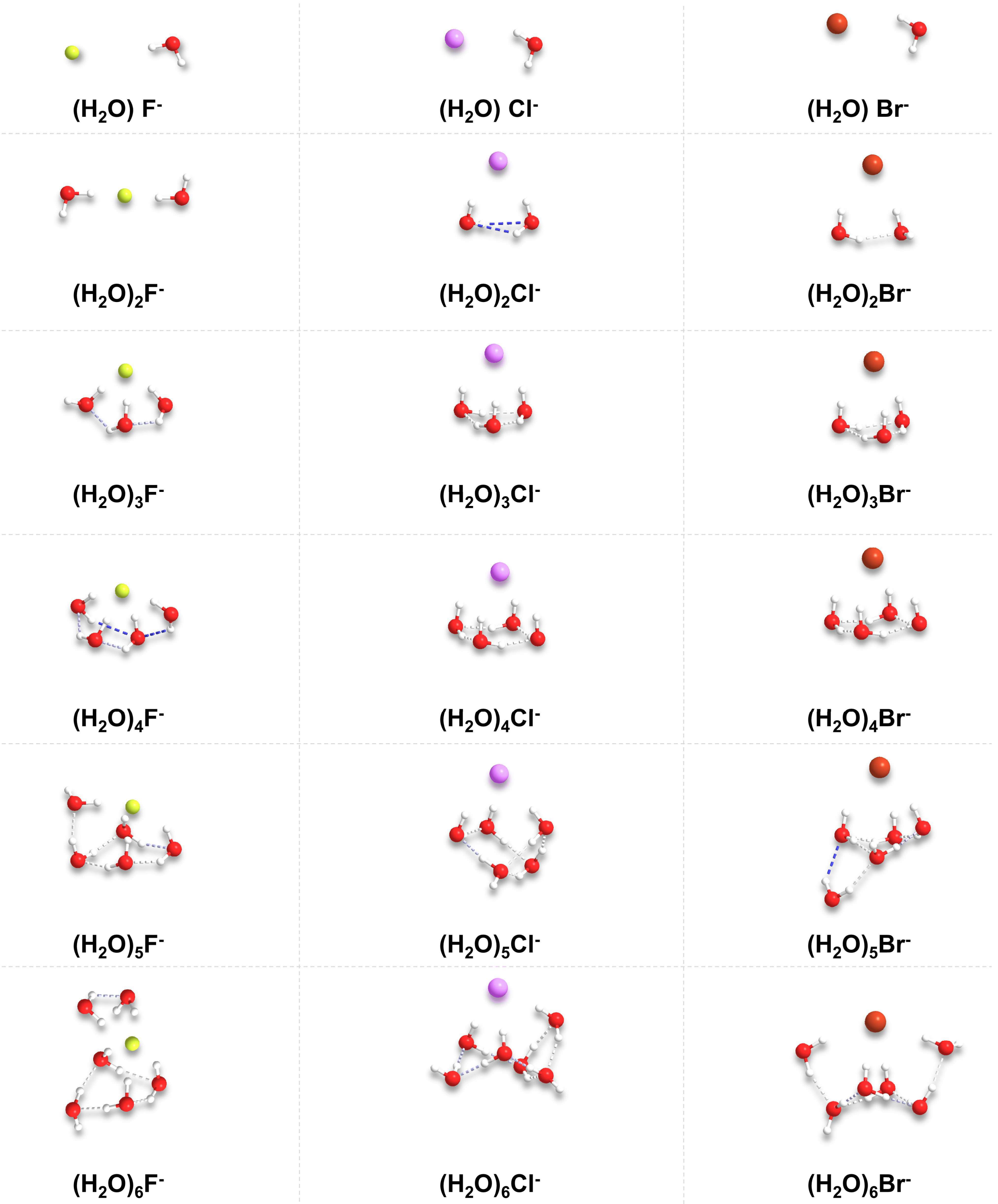

Fig. S4. Structures of $(H_2O)_nX^-$ (X=F, Cl and Br, n=1-6) calculated using *ab initio* DFT. Red and white balls denote oxygen and hydrogen atoms. Yellow, purple and dark red balls represent fluoride ions, chloride ions and bromine ions, respectively. The results indicate that $(H_2O)_nF^-$ favors internal/semi-internal structures, while $(H_2O)_nCl^-$ and $(H_2O)_nBr^-$ favor surface structures, which are consistent with previous work (*31, 32*). The internal/semi-internal hydration structures are more symmetrically arranged compared to the surface structures. This results in $(H_2O)_nF^-$ being more sensitive to the confinement of steric effect.

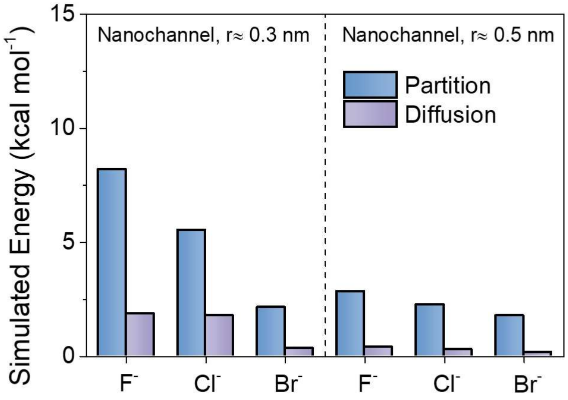

Fig. S5. Simulated energy for hydrated anions entering the polyamide nanochannels (i.e., partitioning) and diffusing inside (i.e., intrapore diffusion) calculated from PMF. More detailed information for calculating energy can be found in Fig. 2C of the manuscript.

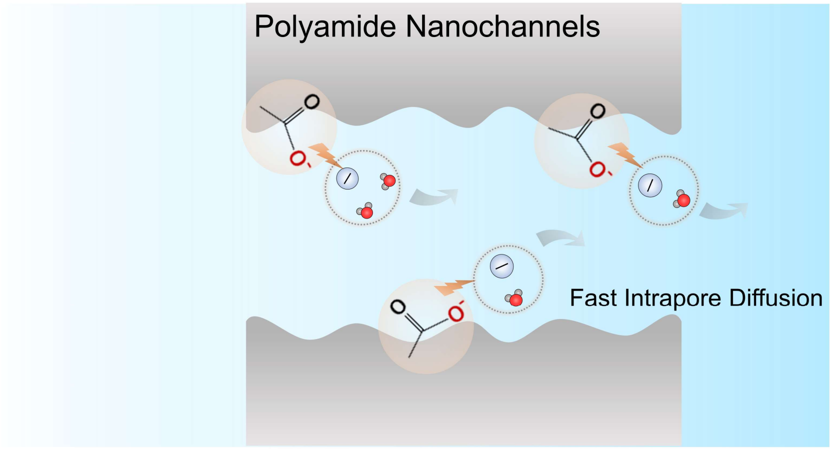

Fig. S6. Schematic illustrations showing the intrapore diffusion of anion in the polyamide nanochannels. In our experimental conditions (pH ≥ 6.0), carboxyl groups on the internal surface of nanochannels in polyamide membranes are negatively ionized, which results in negatively charged internal surface of nanochannels. The charge repulsion between negatively charged carboxyl groups and anions is favorable for anions to bypass the functional sites (carboxyl groups) and fast transport, which produces relatively small hindrance, i.e., lower energy from intrapore diffusion. Hence, such fast intrapore diffusion of anions in polyamide nanochannels hardly contributes to the overall apparent transmembrane energy barriers (E_a) (*4, 11, 18, 62*), showing that partitioning into the nanochannels should contribute higher resistance for the permeation of solvated anions through polyamide nanochannels.

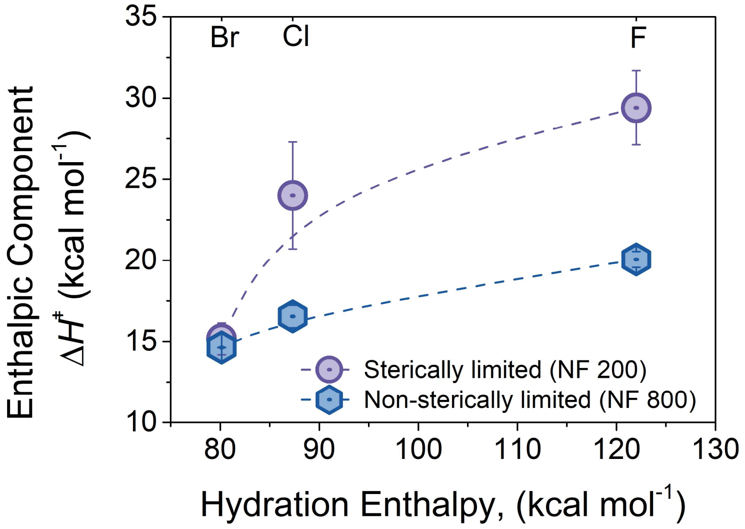

Fig. S7. Comparison between the hydration enthalpy of anions and the enthalpic component ($\Delta H^{\ddagger}$) of apparent transmembrane energy for anions transport through the sterically limited membrane (NF 200) and the non-sterically limited membrane (NF 800).

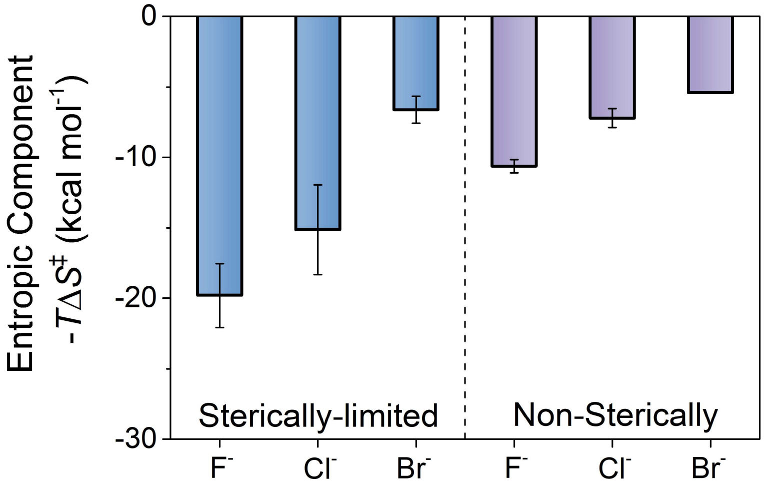

Fig. S8. The entropic component ($-T\Delta S^{\ddagger}$) at 298.15K of apparent transmembrane energy for the anions transport through the sterically limited NF 200 (blue column) and the non-sterically limited NF 800 (purple column) membranes.

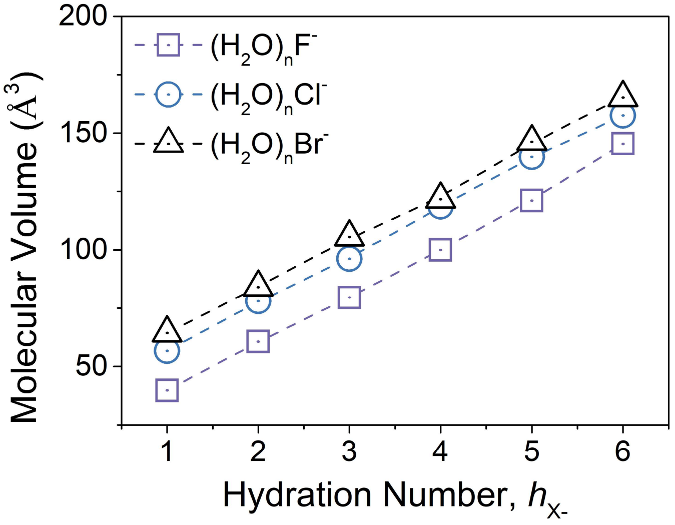

Fig. S9. The molecular volume of hydrates with different hydration number calculated by density functional theory (DFT).

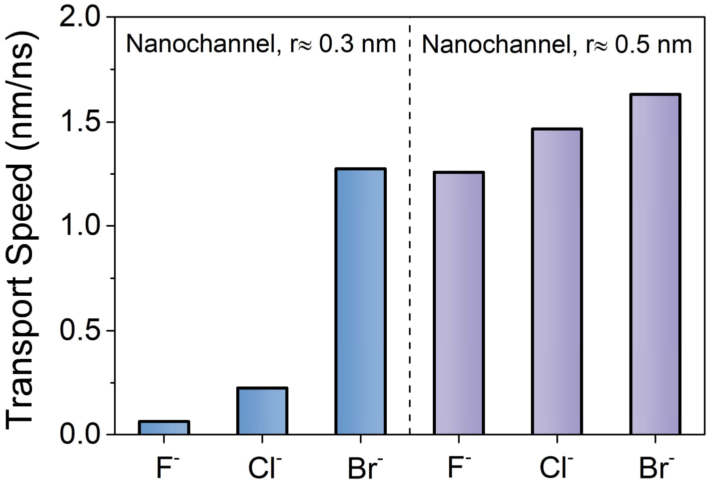

Fig. S10. The transport speed of different hydrates during simulated transport through polyamide nanochannels with different size.

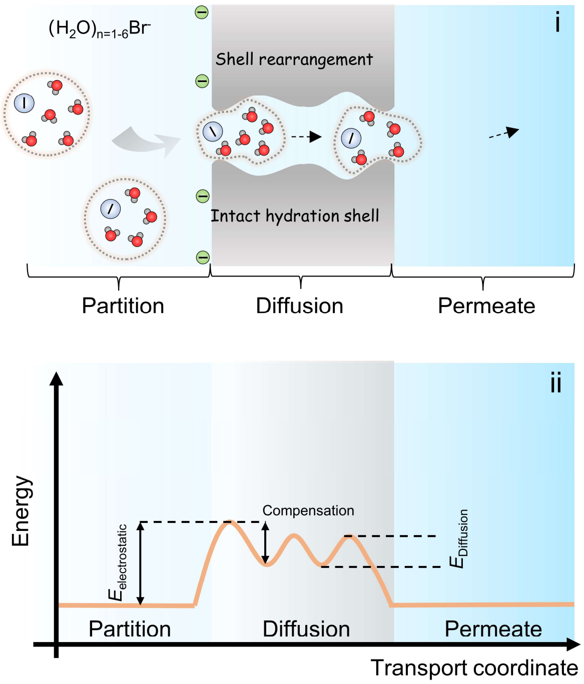

Fig. S11. Schematic diagram showing ion transport and energy profile of weakly hydrated anions for elucidating the mechanisms of dehydration-enhanced electrostatic interactions. Hydrates permeate across the membrane as a result of partitioning into the active layer and diffusion through the active layer. For weakly hydrated anions (e.g., $(H_2O)_nBr^-$), they may rearrange the hydration shell and maintain an intact hydrated structure during transport (i), which contributes lower energy barriers (ii). Light blue and green balls represent the bromide (Br^-) and negative carboxyl, respectively.

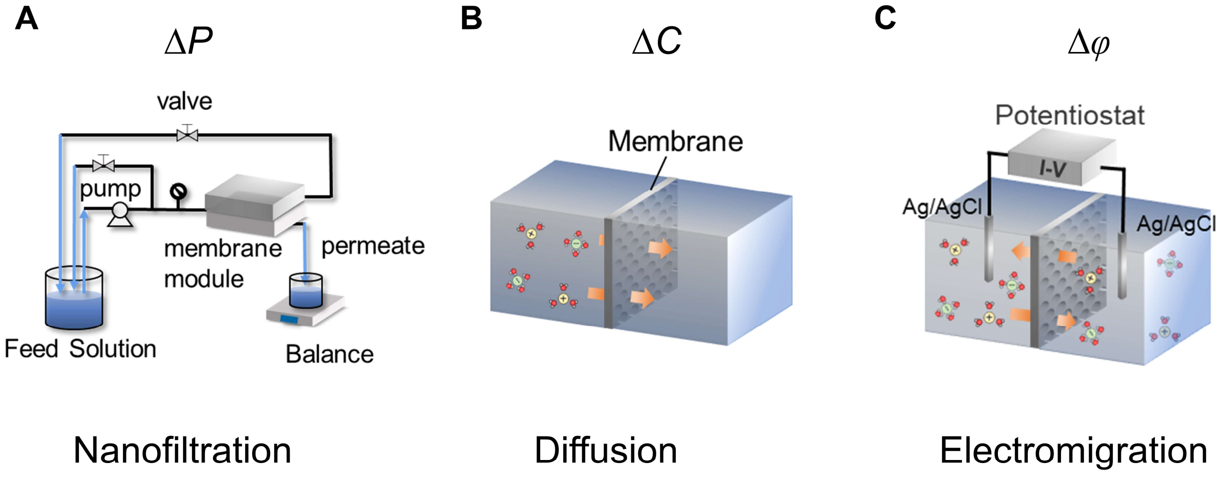

Fig. S12. Schematic diagram of the filtration devices used for anions transport measurements. Anions transport driven by **(A)** pressure (nanofiltration), **(B)** concentration-gradient (diffusion) and **(C)** electric-field (electromigration), respectively.

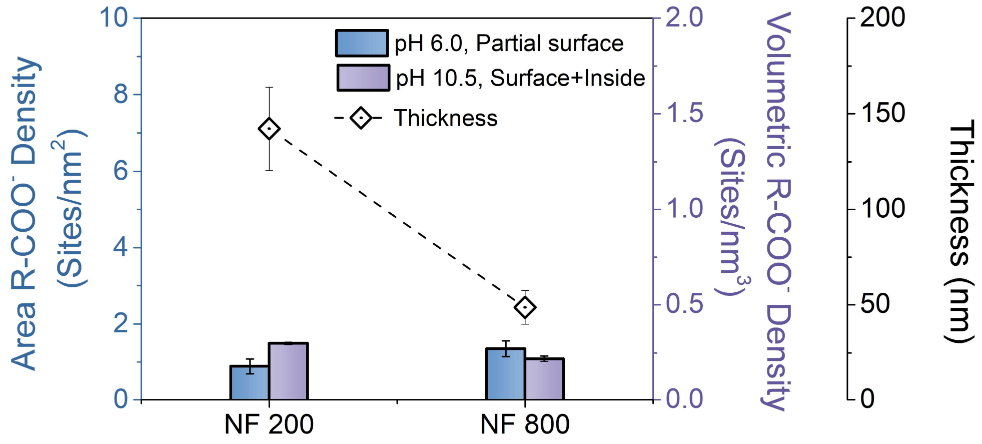

Fig. S13. The ionization behavior of carboxyl in polyamide membranes. At pH 6.0, only partial surface carboxyl groups were ionized in polyamide membranes, the carboxyl density was given by the areal R-COO$^-$ density (blue columns). When pH was increased to 10.5, all carboxyl groups, including inside of the polyamide membrane, were completely ionized, and the carboxyl group density was thus given by the volumetric R-COO$^-$ density (purple columns). The thickness of active layer in polyamide membrane was measured by QCM.

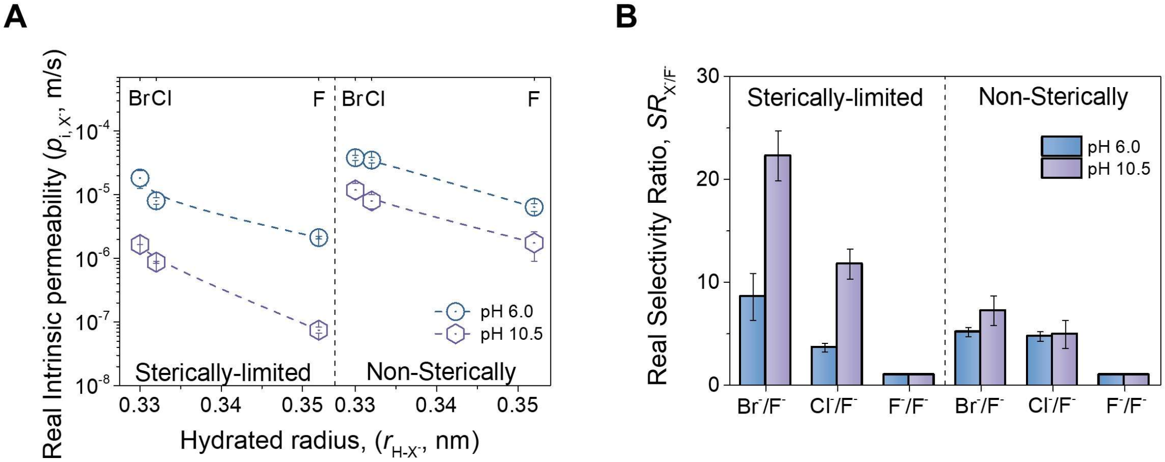

Fig. S14. Anion transport and selectivity of nanoporous polyamide membrane in nanofiltration when accounting for concentration polarization. (A) Real intrinsic permeability of monovalent anions in nanofiltration under different pH. **(B)** Real anion selectivity ratio (SR_{X^-/F^-}) of the two polyamide nanofiltration membranes (i.e., sterically limited NF 200 and non-sterically limited NF 800) under different pH. To determine the real intrinsic permeability after considering concentration polarization, we calculated the mass transfer coefficient at the boundary layer on the membrane according to the film theory. The detailed calculation was provided in *Supplementary Materials* (Note. S11).

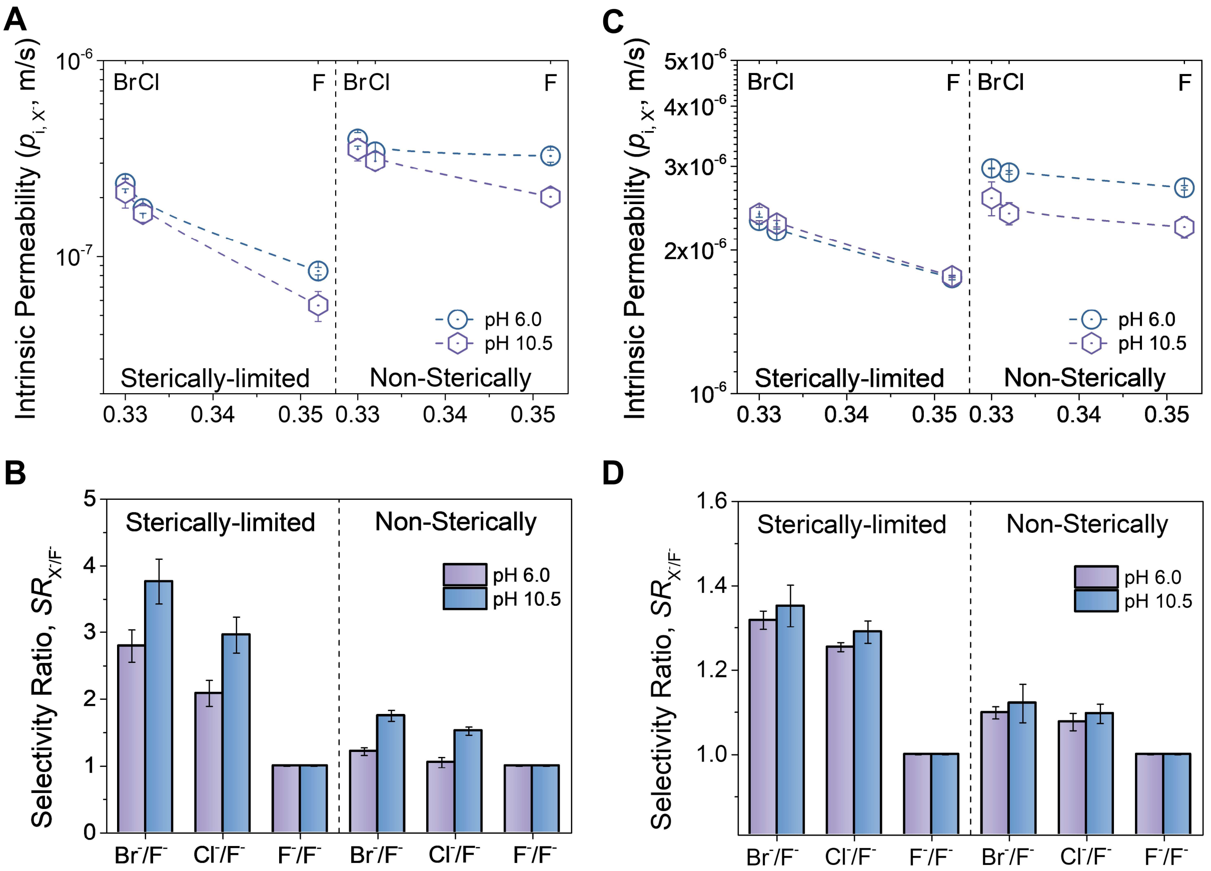

Fig. S15. Anion transport and selectivity of nanoporous polyamide membrane driven by various driving forces at different pH. (A)-(B) Intrinsic permeability and selectivity ratio of monovalent anions (i.e., F^-, Cl^- and Br^-) in polyamide membranes under different pH driven by concentration gradient (diffusion), respectively. **(C)-(D)** Intrinsic permeability and selectivity ratio of monovalent anions in polyamide membranes under different pH driven by electric field (electromigration), respectively.

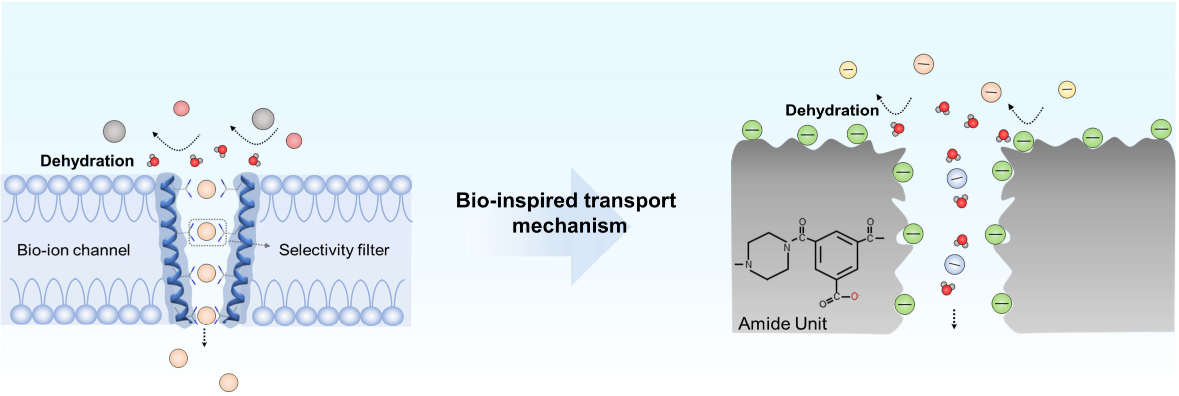

Fig. S16. Bioinspired ion transport mechanisms of nanoporous polymeric nanochannels. Biological ion channel, such as KcsA K^+ channel, is an exquisite instance of ultra-high ion selectivity achieved through rational combination between dehydration and solute-channels interactions (*7, 21, 22, 72*). Specifically, with well-tailored asymmetrical nanochannel morphology, KcsA K^+ channel can adjust size of hydrated K^+ by dehydration, allowing K^+ to more energy-favorably interact with the binding sites and access into channel. Then a strong electrostatic-interactions between the dehydrated K^+ drives ultrafast K^+ conduction. Other ions such as Na^+, are less energetically favorable to interact with the binding sites. This mechanism leads to a K^+/Na^+ selectivity up to 10^4 (*5, 36, 73*). In our study, although the structure of the polymeric membrane is less sophisticated than that of biological ion channels, the ion transport and selectivity are governed by the structure matching-based synergy between pore size and functional group, which highlights the dehydration enhanced charge repulsion.

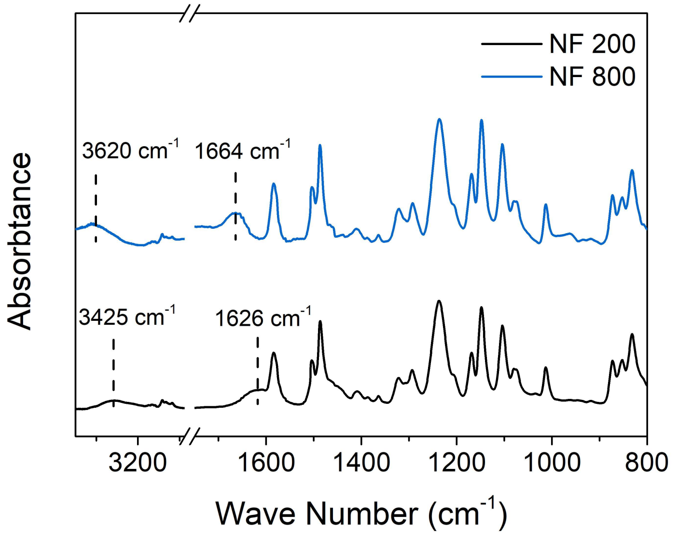

Fig. S17. ATR-FTIR spectra of the studied polyamide membranes. NF 200 and NF 800 exhibited typical Poly(piperazineamide) based semi-aromatic polyamide characteristics (*74*). The absorbed peaks at 1664 cm^{-1} and 1626 cm^{-1} are attributed to C=O stretching, C-N stretching, and C-C-N deformation vibration, which indicates the amide I band (*74*). The broad band at around 3500 cm^{-1} is derived from N-H stretching (*74*) and the -OH stretching of carboxylic acid groups formed by the partial hydrolysis of the trimesoyl chloride (*75*).The R–COOH is considered as the dominant ionizable group under the pH range used in our study (pH ≥ 6.0) (*58, 76*).

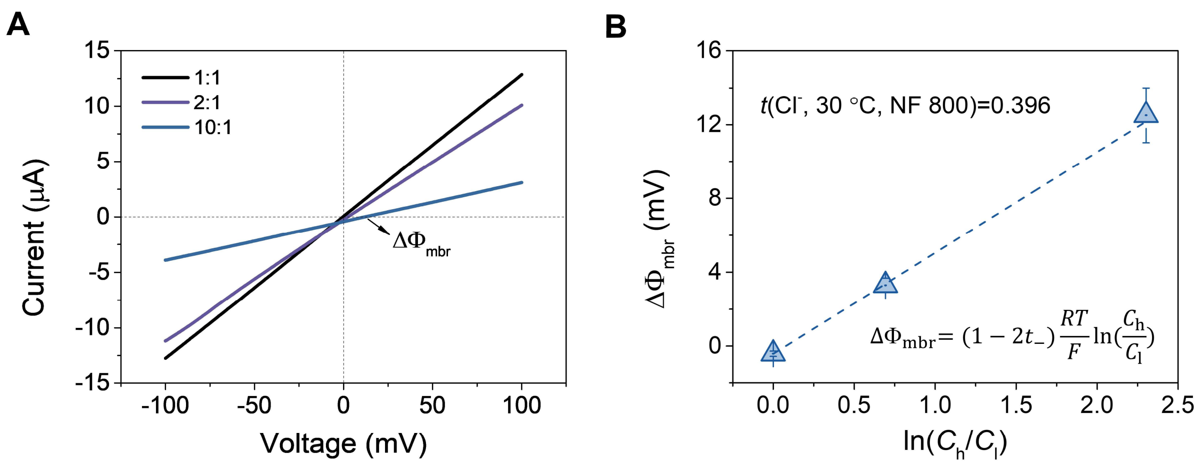

Fig. S18. Measurement of the anions transport number (t_-) across the polyamide membranes. (A) *I-V* curves of the NaX solutions with different $\frac{C_{\text{h}}}{C_{\text{l}}}$ during transport through the polyamide membranes were obtained by the LSV technique. *I-V* curves of NaCl across NF 800 membrane at 30 ºC are shown here as an example. The $\frac{C_h}{C_l}$ were 1:1 (10mM :10 mM), 2:1 (10mM :5 mM) and 10:1 (10mM :1 mM). The horizontal intercept (voltage, mV) of the *I-V* curve represents the membrane potential ($\Delta\Phi_{\text{mbr}}$). The *I-V* curves showed a very good linear relationship, which indicates that the *G* was constant throughout the measurement. It can be concluded that from the beginning of the electro-driven process to the end of the test, no influence of concentration polarization on the ionic flux. Therefore, the concentration polarization can be ignored in the measurement of apparent transmembrane energy barriers by the electro-driven method. **(B)** Linear dependence of the membrane potential ($\Delta\Phi_{\text{mbr}}$) on the concentration ratio (ln $(\frac{C_{\text{h}}}{C_{\text{l}}})$) between the two chambers of the electro-driven apparatus. The anions transport number (t_-) was thus calculated with the slope of the linear equation.

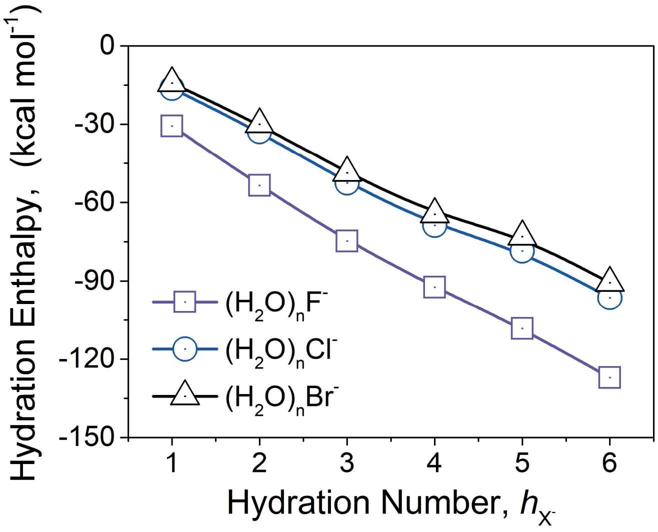

Fig. S19. The hydration enthalpy of hydrates with different hydration number calculated by density functional theory (DFT).

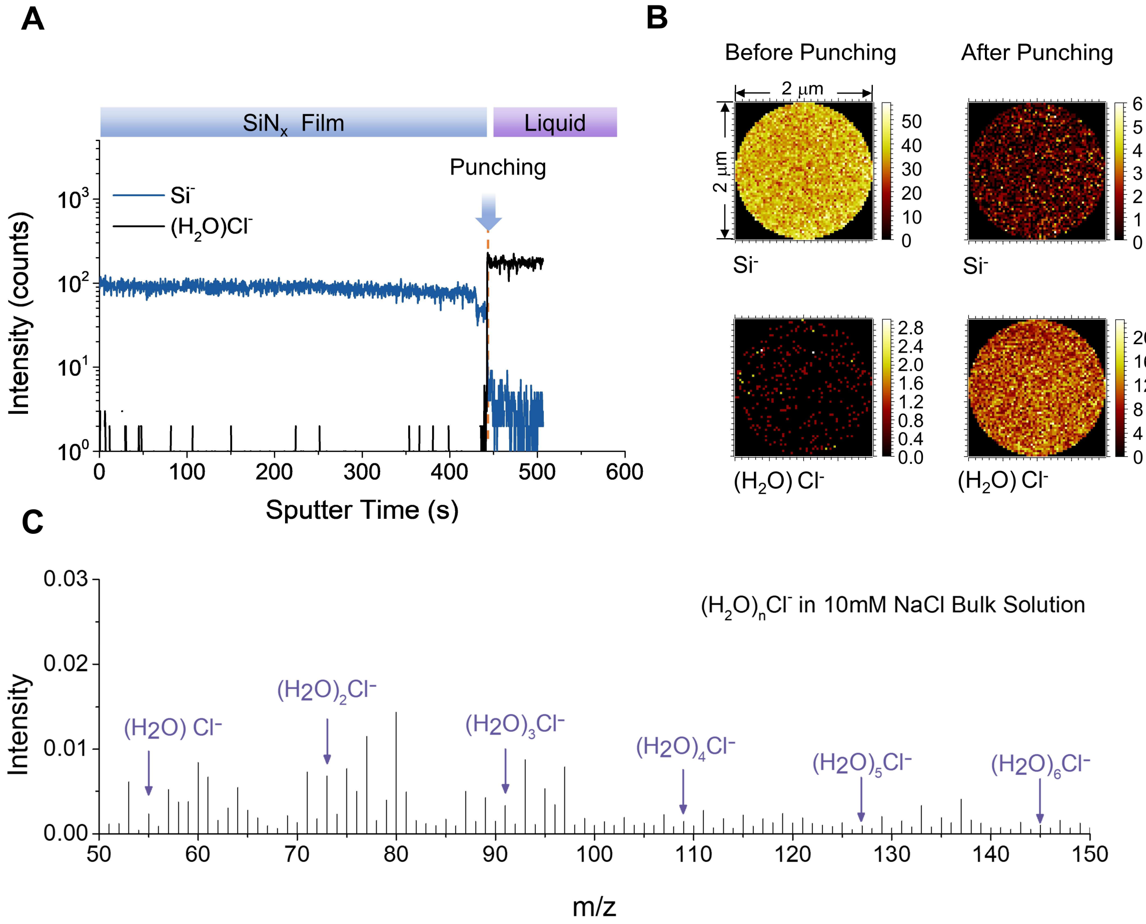

Fig. S20. Discrimination of the hydration distribution during transmembrane ion transport by the *in situ* liquid ToF-SIMS. (A) Representative dynamic ToF-SIMS depth profiling of Si^- and (H_2O) Cl^-. The Si^- was from the SiN_x film and (H_2O) Cl^- was from 10 mM NaCl solution. The SiN_x film was punched through at around 445 s, allowing NaCl solution to permeate through the polyamide membrane. We thus observed that the Si^- signals were decreased and (H_2O) Cl^- signals were increased. **(B)** Reconstructed 2D chemical images of Si^- and (H_2O) Cl^- before and after perforation of the SiN_x film. A brighter color represents a stronger signal intensity. The instantaneous mass spectrum information of solution was obtained from this round area of 2 μm in diameter. **(C)** Representative ToF-SIMS spectra of 10 mM NaCl before filtration (i.e., bulk solution) in negative mode. Signal intensities were normalized by total ion intensity statistics. The mass spectrum peaks of $(H_2O)_nCl^-$ (n=1-6) were assigned by their mass-to-charge ratio (m/z), which were marked with purple arrows.

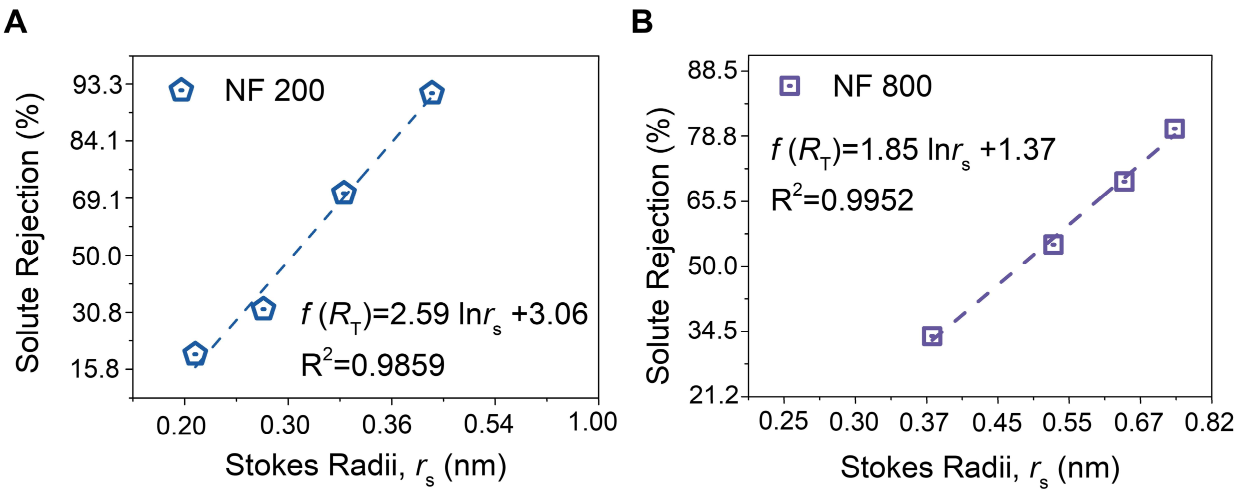

Fig. S21. The membrane effective rejection curves (solute rejection vs. Stokes radii) plotted on a log-normal probability coordinate system. (A) NF 200. **(B)** NF 800. Experiments were conducted under 6 bar of applied pressure. The linearly dependent coefficients were reasonable with $r^2 > 0.95$. The pore size distributions (PSDs) can be calculated by using this linear equation according to Eq. S3.

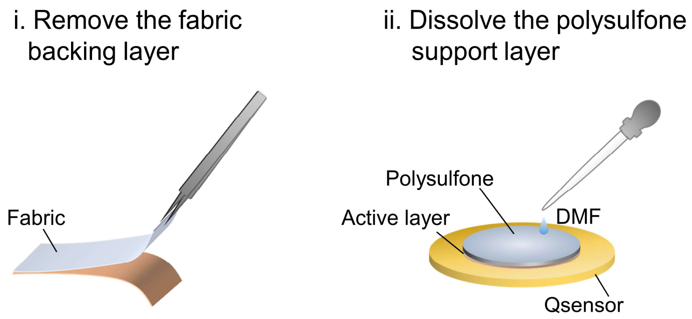

Fig. S22. Schematic illustration of the procedure for the preparation of QCM sample. In brief, **(i)** the fabric backing of membrane coupon was firstly peeled off leaving the polysulfone support layer and polyamide active layer. **(ii)** The polysulfone support layer on the isolated two-layered membrane was then dissolved by *N*, *N*-Dimethylformamide (DMF), leaving the active layer of polyamide membrane on the Qsensor. More details in Note. S8.

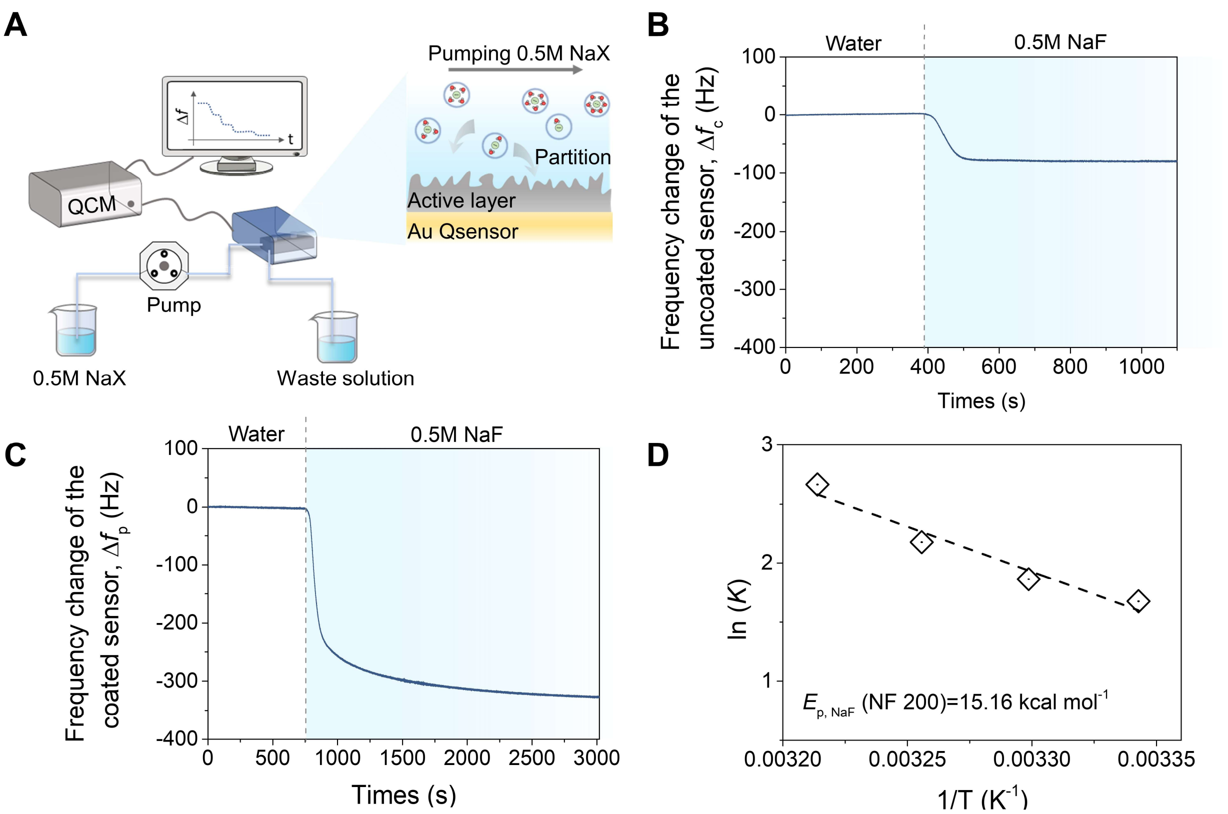

Fig. S23. Measurement of the energy required for NaX salt partitioning into polyamide membranes. (A) Schematic illustration of the QCM measurement. **(B)-(C)** Representative frequency changes in QCM measurements of **(B)** sensor uncoated polyamide membrane (i.e., control sensor) and **(C)** sensor coated polyamide membrane. The solution pH was 6.0 in all experiments. We obtained the real frequency change in salt partitioning ($\Delta f_{\mathrm{p,a}}$) by subtracting the Δf_{c} from the Δf_{p}, i.e., $\Delta f_{\mathrm{p,a}} = \Delta f_{\mathrm{p}} - \Delta f_{\mathrm{c}}$. The $\Delta f_{\mathrm{p,a}}$ at different temperature were measured for calculating the salt partitioning coefficient (*K*). **(D)** Arrhenius-type plots for NaX salt partitioning into polyamide membranes. With the calculated *K* at different temperature, the energy required for NaX salt partitioning into polyamide membranes can be determined by the Arrhenius-type equation (Eq. S8).

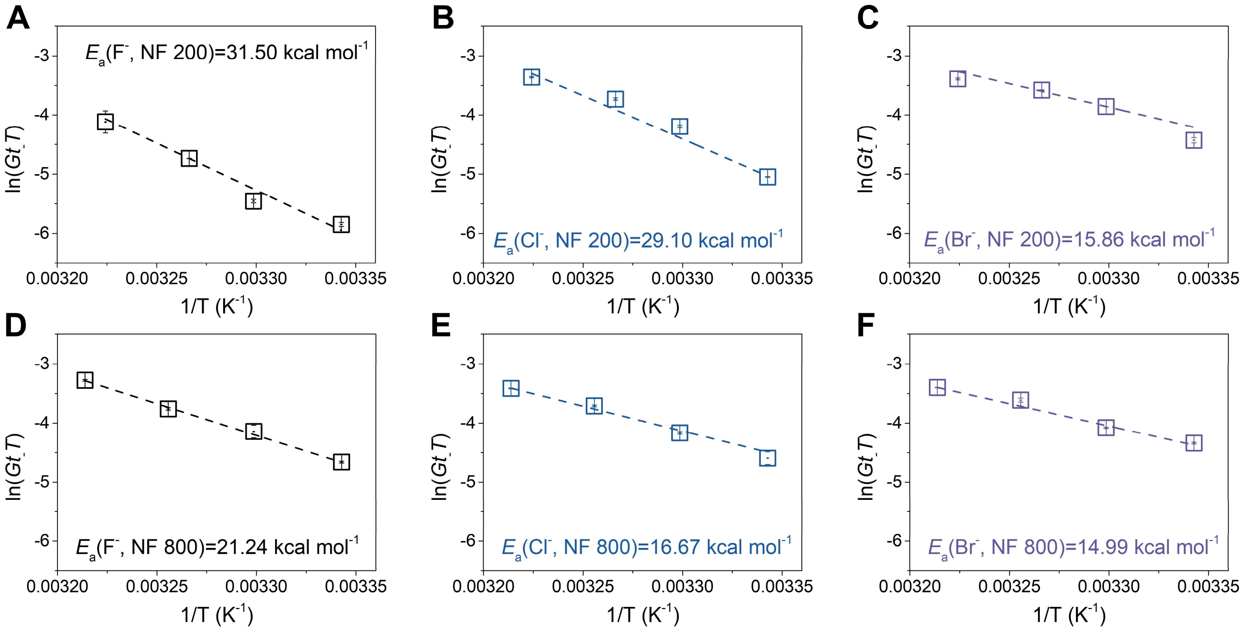

Fig. S24. Arrhenius-type plots for anions transport through polyamide membranes. (A)-(C) F^-, Cl^- and Br^- transport through NF 200. **(D)-(F)** F^-, Cl^- and Br^- transport through NF 800. The overall ionic conductance (*G*) was determined by the LSV technique in electro-driven ion transport experiments at different temperatures (i.e., 26, 30, 34 and 38 ºC). The transport number (t_-) at corresponding temperature was obtained by measuring the membrane potential (Fig. S18).

Table. S1

Ionic radii (r_{X^-}), hydrated radii (r_{H-X^-}), hydration number (h_{X^-}), hydration enthalpy ($\Delta hydH^*$) and hydration entropy ($\Delta hydS^*$) of the anions investigated in this study.

**Anion**	r_{X^-}[a] (nm)	r_{H-X^-}[a] (nm)	h_{X^-}[b]	$\Delta hydH^*$[b] (kJ mol^{-1})	$\Delta hydS^*$[b] (J K^{-1} mol^{-1})
Fluoride (F^-)	0.136	0.352	2.7	-510	-156
Chloride (Cl^-)	0.181	0.332	2.0	-365	-94
Bromide (Br^-)	0.195	0.330	1.8	-335	-78

Note: All data were collected from the same source whenever possible. [a] From ref (*28*). [b] From ref (*59*). The hydrated radii (r_{H-X^-}) were estimated by corrections to the Stokes radii (*28*).

Table. S2

Characteristic parameters in the probability density functions for polyamide membranes investigated in this study.

Membrane	Fitted equation	Adj.R^2	μ_p (nm)	σ_p
NF 200	$f(R_T)=2.59\ln r_s+3.06$	0.9859	0.30	1.47
NF 800	$f(R_T)=1.85\ln r_s+1.37$	0.9952	0.48	1.71

Table. S3

The diffusion coefficient (D_W) of the anions in dilute aqueous solution at different temperature (*77, 78*).

Anions	D_W, 10^{-5} cm^2 s^{-1}				
	**15 ºC**	**18 ºC**	**25 ºC**	**35 ºC**	**45 ºC**
F^-	-	1.230	1.475	-	-
Cl^-	1.580	1.716	2.033	2.538	3.095
Br^-	1.625	1.768	2.081	2.588	3.145

Table. S4

The proportion (P_j, %) of ($H_2O)_nX^-$ (n=1-6) with j configuration (Config.) calculated by the Boltzmann distribution law based on relative binding energies (ΔE_j, kcal mol^{-1}).

h_{X^-}	Config.	$-\Delta E_0$	ΔE_j	$Q_{j(\mathrm{Relat})}$	P_j (%)
			$(H_2O)_nF^-$		
1	1(C_s)	25.1	0	1	99.998
	1(C_{2v})	18.6	6.5	1.71×10^{-5}	0.002
2	2(C_2)	43.4	0.8	0.23	16.50
	2(C_{2v})	34.4	9.8	1.42×10^{-8}	0.000001
	2(C_{2h})	44.2	0	1	72.10
	2(C'_{2v})	43.2	1	0.16	11.41
3	3(C$_3$)	58.3	0.3	0.58	36.24
	3(C_{3h})	58.6	0	1	63.01
	2+1(C_s)	56.2	2.4	0.012	0.75
	4(C_4)	68.9	3.7	0.0011	0.11
	3+1(C_s)	69.6	3	0.0040	0.38
4	4(C_1)	70.1	2.5	0.010	0.96
	4(C_i)	70.2	2.4	0.012	1.16
	4(C_{4h})	72.6	0	1	96.44
	4(C_2)	70.1	2.5	0.010	0.96
5	L3DL	85.32	0	1	54.50
	L3L2	84.47	0.85	0.21	11.37
	R4A	84.75	0.57	0.35	19.06
	R3AA	84.52	0.8	0.23	12.47
	R43f	83.67	1.65	0.048	2.60
6	R4AA	97.27	0	1	69.32
	R3AAL	96.44	0.83	0.22	15.01
	Bf	96.09	1.18	0.11	7.87
	Bf′	95.88	1.39	0.077	5.35
	R3Ada	95.38	1.89	0.031	2.13
	L3L3	94.36	2.91	0.0047	0.32
			$(H_2O)_nCl^-$		
1	1(C_s)	13.1	0	1	84.41
	1(C_{2v})	12.1	1	0.18	15.59
2	2(C_1)	25.8	0	1	39.00
	2(C_2)	25.7	0.1	0.83	32.43
	2(C_{2v})	22.8	3	0.0040	0.15
	2(C_{2h})	25.3	0.5	0.40	15.51
	2(C'_{2v})	25.2	0.6	0.33	12.90
3	3(C_3)	36.2	0	1	68.48
	3(C_{3h})	35	1.2	0.11	7.49

	2+1(C_s)	35.5	0.7	0.28	18.84
	3+0(C'_s)	34.8	1.4	0.076	5.18
4	4(C_4)	46.8	0	1	86.11
	3+1(C_s)	44.7	2.1	0.021	1.79
	4(C'_1)	45.4	1.4	0.076	6.52
	4(C_i)	44.9	1.9	0.030	2.59
	4(C_{4h})	44.6	2.2	0.017	1.49
	4(C_2)	44.6	2.2	0.017	1.49
5	R4A	57.99	0	1	88.60
	R43f	56.75	1.24	0.10	9.00
	R4f3	55.6	2.39	0.012	1.08
	R3AA	55.58	2.41	0.012	1.04
	R3AA'	54.66	3.33	0.0022	0.19
	R5	54.18	3.81	0.00089	0.079
6	Bf′	67.65	0	1	37.40
	Bf	67.53	0.12	0.80	29.98
	R4AA	67.37	0.28	0.60	22.32
	Bd′	66.92	0.73	0.26	9.74
	Bd	64.96	2.69	0.0070	0.26
	R53f	64.82	2.83	0.0054	0.20
	Bgf	64.13	3.52	0.0015	0.057
	R6	63.45	4.2	0.00043	0.016
	R3AAA	63.41	4.24	0.00040	0.015
			$(H_2O)_nBr^-$		
1	1(C_s)	11.5	0	1	69.94
	1(C_{2v})	11	0.5	0.430	30.06
2	2(C_1)	22.1	0	1	38.79
	2(C_2)	21.9	0.2	0.69	26.83
	2(C_{2v})	20.8	1.3	0.091	3.53
	2(C_{2h})	21.6	0.5	0.40	15.43
	2(C'_{2v})	21.6	0.5	0.40	15.43
3	3(C_3)	32.9	0	1	79.75
	3(C_{3h})	30.9	2	0.025	2.00
	2+1(C_s)	32.1	0.8	0.23	18.25
4	4(C_4)	43.3	0	1	87.33
	4(C_2)	40	3.3	0.0023	0.20
	4(C'_1)	41.3	2	0.025	2.19
	4(C_i)	39.9	3.4	0.0019	0.17
	4(C_{4h})	39.3	4	0.00063	0.055
	3+1(C_s)	41.7	1.6	0.052	4.57
	3+1(C'_1)	41.8	1.5	0.063	5.50
5	R4A	54.11	0	1	87.52

	R43f	53.02	1.09	0.13	11.73
	R4f3	51.42	2.69	0.0070	0.61
	R5	50.2	3.91	0.00074	0.065
	R3AA′	50.19	3.92	0.00073	0.063
6	Bf′	63.69	0	1	37.44
	Bf	63.51	0.18	0.72	26.87
	Bd′	63.37	0.32	0.55	20.75
	Bd	63.18	0.51	0.39	14.62
	R4AA	60.72	2.97	0.0042	0.16
	R53f	60.56	3.13	0.0031	0.12
	Bgf	59.86	3.83	0.00086	0.032
	R6	59.3	4.39	0.00031	0.011
	R3AAA	58.75	4.94	0.00011	0.0041

Note: The h_{X^-} represents hydration number. The relative binding energies (ΔE_j) are the binding energy difference between the j configuration and the minimum energy structure. The binding energies (ΔE_0, kcal mol^{-1}) of all hydrates with various configurations were from ref (*31, 32*). As shown in Table. S4, the most probable geometries for $(H_2O)_nF^-$ were 1(C_s), 2(C_{2h}), 3(C_{3h}), 4(C_{4h}), L3DL and R4AA. For $(H_2O)_nCl^-$ and $(H_2O)_nBr^-$, the most probable geometries were 1(C_s), 2(C_1), 3(C_3), 4(C_4), R4A and Bf'/Bf. Therefore, we used these configurations to analyze the influence of geometry on the transport behavior of hydrates (Fig. S4).

Table. S5

The geometric parameters of $(H_2O)_nX^-$.

h_{X^-}	HBs	r_{X-H} (Å)	r_{O-H_X}, (Å)	r_{O-H_W}, (Å)
		$(H_2O)_nF^-$		
1	0	1.2890	1.1000	0.9590
2	0	1.5105	1.0165	0.9690
3	2	1.6017	1.0020	0.9730
4	4	1.6802	0.9915	0.9747
5	5	1.6893	0.9937	0.9814
6	5	1.7426	0.9866	0.9807
		$(H_2O)_nCl^-$		
1	0	2.1510	0.9600	0.9870
2	2	2.3095	0.9815	0.9710
3	3	2.4413	0.9790	0.9840
4	4	2.5533	0.9770	0.9910
5	6	2.3387	0.9810	0.9924
6	7	2.2703	0.9823	0.9934
		$(H_2O)_nBr^-$		
1	0	4.0360	0.9420	0.9420
2	1	2.4485	0.9820	0.9750
3	3	2.4983	0.9810	0.9840
4	4	2.5693	0.9780	0.9900
5	6	2.4810	0.9820	0.9880
6	6	2.4153	0.9808	0.9891

Note: The HBs represent the water-water hydrogen bond numbers between bound water molecules in $(H_2O)_nX^-$. r_{X-H} is the distance between the X^- and water molecules. r_{O-H_X} represents the O-H bond length of water molecules interacting with X^-, and r_{O-H_w} denotes the O-H bond length of water molecules interacting with neighboring water. All geometric parameters are average values.

REFERENCES AND NOTES

1. M. A. Shannon, P. W. Bohn, M. Elimelech, J. G. Georgiadis, B. J. Marinas, A. M. Mayes, Science and technology for water purification in the coming decades. *Nature* **452**, 301–310 (2008).

2. K. Wang, X. Wang, B. Januszewski, Y. Liu, D. Li, R. Fu, M. Elimelech, X. Huang, Tailored design of nanofiltration membranes for water treatment based on synthesis-property-performance relationships. *Chem. Soc. Rev.* **51**, 672–719 (2022).

3. L. A. Richards, A. I. Schafer, B. S. Richards, B. Corry, The importance of dehydration in determining ion transport in narrow pores. *Small* **8**, 1701–1709 (2012).

4. C. Lu, C. Hu, C. L. Ritt, X. Hua, J. Sun, H. Xia, Y. Liu, D. W. Li, B. Ma, M. Elimelech, J. Qu, In situ characterization of dehydration during ion transport in polymeric nanochannels. *J. Am. Chem. Soc.* **143**, 14242–14252 (2021).

5. Y. F. Zhou, J. H. Morais-Cabral, A. Kaufman, R. MacKinnon, Chemistry of ion coordination and hydration revealed by a K^+ channel-Fab complex at 2.0 Å resolution. *Nature* **414**, 43–48 (2001).

6. S. Y. Noskov, S. Berneche, B. Roux, Control of ion selectivity in potassium channels by electrostatic and dynamic properties of carbonyl ligands. *Nature* **431**, 830–834 (2004).

7. B. Roux, R. MacKinnon, The cavity and pore helices in the KcsA K^+ channel: Electrostatic stabilization of monovalent cations. *Science* **285**, 100–102 (1999).

8. R. Epsztein, R. M. DuChanois, C. L. Ritt, A. Noy, M. Elimelech, Towards single-species selectivity of membranes with subnanometre pores. *Nat. Nanotechnol.* **15**, 426–436 (2020).

9. I. Shefer, K. Lopez, A. P. Straub, R. Epsztein, Applying transition-state theory to explore transport and selectivity in salt-rejecting membranes: A critical review. *Environ. Sci. Technol.* **56**, 7467–7483 (2022).

10. C. L. Ritt, M. Liu, T. A. Pham, R. Epsztein, H. J. Kulik, M. Elimelech, Machine learning reveals key ion selectivity mechanisms in polymeric membranes with subnanometer pores. *Sci. Adv.* **8**, eabl5771 (2022).

11. R. Epsztein, E. Shaulsky, M. Qin, M. Elimelech, Activation behavior for ion permeation in ion-exchange membranes: Role of ion dehydration in selective transport. *J. Membr. Sci.* **580**, 316–326 (2019).

12. B. Tansel, Significance of thermodynamic and physical characteristics on permeation of ions during membrane separation: Hydrated radius, hydration free energy and viscous effects. *Sep. Purif. Technol.* **86**, 119–126 (2012).

13. R. Epsztein, E. Shaulsky, N. Dizge, D. M. Warsinger, M. Elimelech, Role of ionic charge density in donnan exclusion of monovalent anions by nanofiltration. *Environ. Sci. Technol.* **52**, 4108–4116 (2018).

14. V. Pavluchkov, I. Shefer, O. Peer-Haim, J. Blotevogel, R. Epsztein, Indications of ion dehydration in diffusion-only and pressure-driven nanofiltration. *J. Membr. Sci.* **648**, 120358 (2022).

15. L. A. Richards, B. S. Richards, B. Corry, A. I. Schafer, Experimental energy barriers to anions transporting through nanofiltration membranes. *Environ. Sci. Technol.* **47**, 1968–1976 (2013).

16. X. Zhou, Z. Wang, R. Epsztein, C. Zhan, W. Li, J. D. Fortner, T. A. Pham, J. H. Kim, M. Elimelech, Intrapore energy barriers govern ion transport and selectivity of desalination membranes. *Sci. Adv.* **6**, eabd9045 (2020).

17. I. Shefer, O. Peer-Haim, O. Leifman, R. Epsztein, Enthalpic and entropic selectivity of water and small ions in polyamide membranes. *Environ. Sci. Technol.* **55**, 14863–14875 (2021).

18. S. B. Sigurdardottir, R. M. DuChanois, R. Epsztein, M. Pinelo, M. Elimelech, Energy barriers to anion transport in polyelectrolyte multilayer nanofiltration membranes: Role of intra-pore diffusion. *J. Membr. Sci.* **603**, 117921 (2020).

19. X. Li, H. Zhang, P. Wang, J. Hou, J. Lu, C. D. Easton, X. Zhang, M. R. Hill, A. W. Thornton, J. Z. Liu, B. D. Freeman, A. J. Hill, L. Jiang, H. Wang, Fast and selective fluoride ion conduction in sub-1-nanometer metal-organic framework channels. *Nat. Commun.* **10**, 2490 (2019).

20. B. Corry, Mechanisms of selective ion transport and salt rejection in carbon nanostructures. *MRS Bull.* **42**, 306–310 (2017).

21. J. Payandeh, T. Scheuer, N. Zheng, W. A. Catterall, The crystal structure of a voltage-gated sodium channel. *Nature* **475**, 353–358 (2011).

22. D. A. Doyle, J. Morais Cabral, R. A. Pfuetzner, A. Kuo, J. M. Gulbis, S. L. Cohen, B. T. Chait, R. MacKinnon, The structure of the potassium channel: Molecular basis of K^+ conduction and selectivity. *Science* **280**, 69–77 (1998).

23. R. B. Schoch, Transport phenomena in nanofluidics. *Rev. Mod. Phys.* **80**, 839–883 (2008).

24. Y. Marcus, Thermodynamics of solvation of ions. Part 5.—Gibbs free energy of hydration at 298.15 K. *J. Chem. Soc. Faraday Trans.* **87**, 2995–2999 (1991).

25. H. J. Bakker, Structural dynamics of aqueous salt solutions. *Chem. Rev.* **108**, 1456–1473 (2008).

26. K. D. Collins, Charge density-dependent strength of hydration and biological structure. *Biophys. J.* **72**, 65–76 (1997).

27. L. A. Richards, A. I. Schafer, B. S. Richards, B. Corry, Quantifying barriers to monovalent anion transport in narrow non-polar pores. *Phys. Chem. Chem. Phys.* **14**, 11633–11638 (2012).

28. E. R. Nightingale, Phenomenological theory of ion solvation. Effective radii of hydrated Ions. *J. Phys. Chem.* **63**, 1381–1387 (1959).

29. J. E. Combariza, N. R. Kestner, J. Jortner, Energy-structure relationships for microscopic solvation of anions in water clusters. *J. Chem. Phys.* **100**, 2851–2864 (1994).

30. S. S. Xantheas, Quantitative description of hydrogen bonding in chloride-water clusters. *J. Phys. Chem. Us* **100**, 9703–9713 (1996).

31. H. M. Lee, D. Kim, K. S. Kim, Structures, spectra, and electronic properties of halide-water pentamers and hexamers, $X^-(H_2O)_{5,6}$ (X=F,Cl,Br,I): Ab initio study. *J. Chem. Phys.* **116**, 5509–5520 (2002).

32. J. Kim, H. M. Lee, S. B. Suh, D. Majumdar, K. S. Kim, Comparative ab initio study of the structures, energetics and spectra of $X^-\cdot(H_2O)_{n=1-4}$ [X=F,Cl,Br,I] clusters. *J. Chem. Phys.* **113**, 5259–5272 (2000).

33. M. Yan, P. He, Y. Chen, S. Wang, Q. Wei, K. Zhao, X. Xu, Q. An, Y. Shuang, Y. Shao, K. T. Mueller, L. Mai, J. Liu, J. Yang, Water-lubricated intercalation in $V_2O_5 \cdot nH_2O$ for high-capacity and high-rate aqueous rechargeable zinc batteries. *Adv. Mater.* **30**, 1703725 (2018).

34. P. Novak, J. Desilvestro, Electrochemical insertion of magnesium in metal oxides and sulfides from aprotic electrolytes. *J. Electrochem. Soc.* **140**, 140–144 (1993).

35. H. Zhang, X. Liu, H. Li, I. Hasa, S. Passerini, Challenges and strategies for high-energy aqueous electrolyte rechargeable batteries. *Angew. Chem. Int. Ed. Engl.* **60**, 598–616 (2021).

36. B. Corry, The naked truth about K^+ selectivity. *Nat. Chem.* **10**, 799–800 (2018).

37. T. Xu, B. Wu, L. Hou, Y. Zhu, F. Sheng, Z. Zhao, Y. Dong, J. Liu, B. Ye, X. Li, L. Ge, H. Wang, T. Xu, Highly ion-permselective porous organic cage membranes with hierarchical channels. *J. Am. Chem. Soc.* **144**, 10220–10229 (2022).

38. Y. Zhang, W. Zeng, L. Huang, W. Liu, E. Jia, Y. Zhao, F. Wang, Z. Zhu, In situ liquid secondary ion mass spectrometry: A surprisingly soft ionization process for investigation of halide ion hydration. *Anal. Chem.* **91**, 7039–7046 (2019).

39. F. G. Smith, W. M. Deen, Electrostatic double-layer interactions for spherical colloids in cylindrical pores. *J. Colloid Interf. Sci.* **78**, 444–465 (1980).

40. P. L. Paine, P. Scherr, Drag coefficients for the movement of rigid spheres through liquid-filled cylindrical pores. *Biophys. J.* **15**, 1087–1091 (1975).

41. W. M. Deen, Hindered transport of large molecules in liquid-filled pores. *Aiche J.* **33**, 1409–1425 (1987).

42. D. M. Malone, J. L. Anderson, Hindered diffusion of particles through small pores. *Chem. Eng. Sci.* **33**, 1429–1440 (1978).

43. M. Allouzi, A. Imbrogno, A. I. Schafer, Energy barriers for steroid hormone transport in nanofiltration. *Environ. Sci. Technol.* **56**, 16811–16821 (2022).

44. S. V. Talekar, Temperature dependence of activation energies for self-diffusion of water and of alkali ions in aqueous electrolyte solutions. A model for ion selective behavior of biological cells. *Int. J. Quantum Chem.* **12**, 459–469 (1977).

45. B. Hess, C. Kutzner, D. van der Spoel, E. Lindahl, GROMACS 4: Algorithms for highly efficient, load-balanced, and scalable molecular simulation. *J. Chem. Theory Comp.* **4**, 435–447 (2008).

46. W. L. Jorgensen, J. Tirado-Rives, Potential energy functions for atomic-level simulations of water and organic and biomolecular systems. *Proc. Natl. Acad. Sci. U.S.A.* **102**, 6665–6670 (2005).

47. L. S. Dodda, J. Z. Vilseck, J. Tirado-Rives, W. L. Jorgensen, 1.14*CM1A-LBCC: Localized bond-charge corrected CM1A charges for condensed-phase simulations. *J. Phys. Chem. B.* **121**, 3864–3870 (2017).

48. L. S. Dodda, I. Cabeza de Vaca, J. Tirado-Rives, W. L. Jorgensen, LigParGen web server: An automatic OPLS-AA parameter generator for organic ligands. *Nucleic Acids Res.* **45**, W331–W336 (2017).

49. J. S. Hub, B. L. de Groot, D. van der Spoel, g_wham—A free weighted histogram analysis implementation including robust error and autocorrelation estimates. *J. Chem. Theory Comput.* **6**, 3713–3720 (2010).

50. U. Essmann, L. Perera, M. L. Berkowitz, T. Darden, H. Lee, L. G. Pedersen, A smooth particle mesh Ewald method. *J. Chem. Phys.* **103**, 8577–8593 (1995).

51. B. Hess, H. Bekker, H. J. C. Berendsen, J. G. E. M. Fraaije, LINCS: A linear constraint solver for molecular simulations. *J. Comput. Chem.* **18**, 1463–1472 (1997).

52. Y. Zhao, D. G. Truhlar, The M06 suite of density functionals for main group thermochemistry, thermochemical kinetics, noncovalent interactions, excited states, and transition elements: Two new functionals and systematic testing of four M06-class functionals and 12 other functionals. *Theor. Chem. Acc.* **120**, 215–241 (2008).

53. K. Walden, M. E. Martin, L. LaBee, M. Provorse Long, Hydration and charge-transfer effects of alkaline earth metal ions binding to a carboxylate anion, phosphate anion, and guanine nucleobase. *J. Phys. Chem. B* **125**, 12135–12146 (2021).

54. M. J. Frisch, G. W. Trucks, H. B. Schlegel, G. E. Scuseria, M. A. Robb, J. R. Cheeseman, G. Scalmani, V. Barone, G. A. Petersson, H. Nakatsuji, X. Li, M. Caricato, A. V. Marenich, J. Bloino, B. G. Janesko, R. Gomperts, B. Mennucci, H. P. Hratchian, J. V. Ortiz, A. F. Izmaylov, J. L. Sonnenberg, Williams, F. Ding, F. Lipparini, F. Egidi, J. Goings, B. Peng, A. Petrone, T. Henderson, D. Ranasinghe, V. G. Zakrzewski, J. Gao, N. Rega, G. Zheng, W. Liang, M. Hada, M. Ehara, K. Toyota, R. Fukuda, J. Hasegawa, M. Ishida, T. Nakajima, Y. Honda, O. Kitao, H. Nakai, T. Vreven, K. Throssell, J. A. Montgomery Jr., J. E. Peralta, F. Ogliaro, M. J. Bearpark, J. J. Heyd, E. N. Brothers, K. N. Kudin, V. N. Staroverov, T. A. Keith, R. Kobayashi, J. Normand, K. Raghavachari, A. P. Rendell, J. C. Burant, S. S. Iyengar, J. Tomasi, M. Cossi, J. M. Millam, M. Klene, C. Adamo, R. Cammi, J. W. Ochterski, R. L. Martin, K. Morokuma, O. Farkas, J. B. Foresman, D. J. Fox, Gaussian 16, Revision A.03: Gaussian. Inc., Wallingford, CT (2016).

55. T. Lu, S. Manzetti, Wavefunction and reactivity study of benzo[a]pyrene diol epoxide and its enantiomeric forms. *Struct. Chem.* **25**, 1521–1533 (2014).

56. R. Epsztein, W. Cheng, E. Shaulsky, N. Dizge, M. Elimelech, Elucidating the mechanisms underlying the difference between chloride and nitrate rejection in nanofiltration. *J. Membr. Sci.* **548**, 694–701 (2018).

57. K. Wang, T. Chung, Fabrication of polybenzimidazole (PBI) nanofiltration hollow fiber membranes for removal of chromate. *J. Membr. Sci.* **281**, 307–315 (2006).

58. C. L. Ritt, J. R. Werber, M. Wang, Z. Yang, Y. Zhao, H. J. Kulik, M. Elimelech, Ionization behavior of nanoporous polyamide membranes. *Proc. Natl. Acad. Sci. U.S.A.* **117**, 30191–30200 (2020).

59. Y. Marcus, A simple empirical-model describing the thermodynamics of hydration of ions of widely varying charges, sizes, and shapes. *Biophys. Chem.* **51**, 111–127 (1994).

60. S. S. Xantheas, L. X. Dang, Critical study of fluoride water interactions. *J. Phys. Chem. Us* **100**, 3989–3995 (1996).

61. D. Chen, J. R. Werber, X. Zhao, M. Elimelech, A facile method to quantify the carboxyl group areal density in the active layer of polyamide thin-film composite membranes. *J. Membr. Sci.* **534**, 100–108 (2017).

62. B. Corry, Designing carbon nanotube membranes for efficient water desalination. *J. Phys. Chem. B* **112**, 1427–1434 (2008).

63. J. Wang, R. S. Kingsbury, L. A. Perry, O. Coronell, Partitioning of alkali metal salts and boric acid from aqueous phase into the polyamide active layers of reverse osmosis membranes. *Environ. Sci. Technol.* **51**, 2295–2303 (2017).

64. M. Rickman, R. H. Davis, J. Pellegrino, Temperature-variation study of neutral solute and electrolyte fractionation through cellulose acetate and polyamide membranes. *J. Membr. Sci.* **461**, 114–122 (2014).

65. R. R. Sharma, S. Chellam, Temperature and concentration effects on electrolyte transport across porous thin-film composite nanofiltration membranes: Pore transport mechanisms and energetics of permeation. *J. Colloid Interface Sci.* **298**, 327–340 (2006).

66. J. Wang, D. S. Dlamini, A. K. Mishra, M. T. M. Pendergast, M. C. Y. Wong, B. B. Mamba, V. Freger, A. R. D. Verliefde, E. M. V. Hoek, A critical review of transport through osmotic membranes. *J. Membr. Sci.* **454**, 516–537 (2014).

67. X. Zhai, Y. L. Wang, R. Dai, X. Li, Z. Wang, Roles of anion-cation coupling transport and dehydration-induced ion-membrane interaction in precise separation of ions by nanofiltration membranes. *Environ. Sci. Technol.* **56**, 14069–14079 (2022).

68. Y. Marcus, Electrostriction, ion solvation, and solvent release on ion pairing. *J. Phys. Chem. B* **109**, 18541–18549 (2005).

69. Y. Marcus, in *Ion Solvation in Neat Solvents in Ions in Solution and Their Solvation* (Wiley, 2015), pp. 107–155.

70. J. O. Mbockris, P. P. S. Saluja, Ionic solvation numbers from compressibilities and ionic vibration potentials measurements. *J. Phys. Chem.* **76**, 2140–2151 (1972).

71. Y. Marcus, The standard partial molar volumes of ions in solution. Part 4. Ionic volumes in water at 0−100 °C. *J. Phys. Chem.* **113**,10285–10291 (2009).

72. B. Hille, C. M. Armstrong, R. MacKinnon, Ion channels: From idea to reality. *Nat. Med.* **5**, 1105–1109 (1999).

73. E. Gouaux, R. Mackinnon, Principles of selective ion transport in channels and pumps. *Science* **310**, 1461–1465 (2005).

74. C. Y. Y. Tang, Y. N. Kwon, J. O. Leckie, Effect of membrane chemistry and coating layer on physiochemical properties of thin film composite polyamide RO and NF membranes. *Desalination* **242**, 149–167 (2009).

75. W. J. L. N. Misdan, A. F. Ismail,T. Matsuura, D. Rana, Study on the thin film composite poly(piperazine-amide) nanofiltration membrane: Impacts of physicochemical properties of substrate on interfacial polymerization formation. *Desalination* **344**, 198–205 (2014).

76. V. Freger, Nanoscale heterogeneity of polyamide membranes formed by interfacial polymerization. *Langmuir* **19**, 4791–4797 (2003).

77. W. M. Haynes, D. R. Lide, T. J. Bruno, *CRC Handbook of Chemistry and Physics* (CRC Press, c1977-, ed. 97, 2016).

78. R. Mills, V. M. M. Lobo, Self-diffusion in electrolyte solutions: A critical examination of data compiled from the literature, in *Physical Sciences Data, vol. 36* (Elsevier, ed. 1, 1989).
